# Spectroscopic capture of a low-spin Mn(IV)-oxo species in Ni–Mn_3_O_4_ nanoparticles during water oxidation catalysis

**DOI:** 10.1038/s41467-020-19133-w

**Published:** 2020-10-16

**Authors:** Sunghak Park, Kyoungsuk Jin, Hyung Kyu Lim, Jin Kim, Kang Hee Cho, Seungwoo Choi, Hongmin Seo, Moo Young Lee, Yoon Ho Lee, Sangmoon Yoon, Miyoung Kim, Hyungjun Kim, Sun Hee Kim, Ki Tae Nam

**Affiliations:** 1 Department of Materials Science and Engineering, Seoul National University, Seoul, 08826 Republic of Korea; 2 Nano Systems Institute, Seoul National University, Seoul, 08826 Republic of Korea; 3 Division of Chemical Engineering and Bioengineering, Kangwon National University, Chuncheon, 24341 Republic of Korea; 4 Western Seoul Center, Korea Basic Science Institute (KBSI), Seoul, 03759 Republic of Korea; 5 Department of Chemistry, Korea Advanced Institute of Science and Technology (KAIST), Daejeon, 34141 Republic of Korea

**Keywords:** Heterogeneous catalysis, Electrocatalysis

## Abstract

High-valent metal-oxo moieties have been implicated as key intermediates preceding various oxidation processes. The critical O–O bond formation step in the Kok cycle that is presumed to generate molecular oxygen occurs through the high-valent Mn-oxo species of the water oxidation complex, i.e., the Mn_4_Ca cluster in photosystem II. Here, we report the spectroscopic characterization of new intermediates during the water oxidation reaction of manganese-based heterogeneous catalysts and assign them as low-spin Mn(IV)-oxo species. Recently, the effects of the spin state in transition metal catalysts on catalytic reactivity have been intensely studied; however, no detailed characterization of a low-spin Mn(IV)-oxo intermediate species currently exists. We demonstrate that a low-spin configuration of Mn(IV), *S* = 1/2, is stably present in a heterogeneous electrocatalyst of Ni-doped monodisperse 10-nm Mn_3_O_4_ nanoparticles via oxo-ligand field engineering. An unprecedented signal (*g* = 1.83) is found to evolve in the electron paramagnetic resonance spectrum during the stepwise transition from the Jahn–Teller-distorted Mn(III). In-situ Raman analysis directly provides the evidence for Mn(IV)-oxo species as the active intermediate species. Computational analysis confirmed that the substituted nickel species induces the formation of a z-axis-compressed octahedral C_4v_ crystal field that stabilizes the low-spin Mn(IV)-oxo intermediates.

## Abstract (web summary)

Understanding reaction intermediates provides a foundation for active electrocatalysts’ design, but it remains elusive for heterogeneous electrocatalysts. Here, the authors report the spectroscopic characterization of low-spin Mn(IV)-oxo as the active intermediates during electrochemical water oxidation.

## Introduction

The identification of intermediate species produced during catalysis provides insight into reaction mechanisms and methods for controlling the underlying reaction pathways. Generally, high-valent transition metal-oxo species are involved in the rate-determining steps of various oxygen atom transfer (OAT) reactions, including C–H bond activation, hydroxylation, and O–O bond formation^1–4^. Characterization and manipulation of high-valent transition metal-oxo species are thus crucial to designing heterogeneous catalysts for water oxidation reaction, where O–O bond formation is the key elementary step. The proposed mechanistic cycle of water oxidation reaction starts with the adsorption of a water molecule on the surface of transition metal oxide catalysts. Then, the adsorbed water molecule undergoes multiple protons and electrons transfer with a redox state change of the transition metal site to generate high-valent transition metal-oxo species involved in the O–O bond formation step. Understanding each elementary step and developing catalysts under neutral pH conditions have been recently getting much interest due to the advantage of environmentally friendly, less corrosive electrolyte and the possibility of combining with CO_2_ reduction or microbial hybrid electrolysis cell^5–7^. While many experimental and computational results imply the existence of a high-valent metal-oxo species on the surface of heterogeneous catalysts that serves as an active reaction intermediate during the water oxidation reaction, very few results offering direct evidence of the chemical identity of intermediates have been reported to date for heterogeneous catalysts.

Various in-situ/ex-situ spectroscopic analyses have been performed on several 1st-row transition metal-based catalysts (Mn, Fe, Co, and Ni) for the characterization of intermediate high-valent metal-oxo species. The presence of Co(IV) species with a low-spin configuration (*S* = 1/2) was demonstrated for cobalt phosphate catalysts during electrochemical water oxidation reaction by ex-situ electron paramagnetic resonance (EPR) analysis^8^. The mechanism of the O–O bond formation step with reactive Co(IV)-oxo (reformulated as a Co(III)-oxyl radical) species is suggested based on the above EPR results, in-situ X-ray absorption spectroscopy results^9,10^, and further isotope labeling studies^11^. Recently, rapid-scan attenuated total reflectance infrared (ATR-IR) spectroscopy was used to identify two distinct Co(IV)-oxo and Co(III)-superoxide species from Co_3_O_4_ nanoparticles (NPs) as reaction intermediates of the photochemical water oxidation reaction^12^. The mechanism involving the Co(III)-superoxide intermediate species leads to a more efficient water oxidation reaction. ATR-IR spectroscopy was also applied to detect the presence of an Fe(IV)-oxo species as an intermediate during photoelectrochemical water oxidation on hematite films^13^, although the precise electronic structure remains elusive. Although there is no experimental data on the direct characterization of the active high-valent metal-oxo species in NiFe oxides/hydroxides at aqueous electrolyte, the results of density functional theory (DFT) calculations suggest that Fe(IV) (high-spin *d*^4^) and Ni(IV) (low-spin *d*^6^) species are active sites for facile oxo formation and favorable O–O bond formation respectively^14^. Interestingly, Gray and colleagues experimentally capture cis-dioxo-Fe(VI) intermediate species from NiFe hydroxide catalyst in a nonaqueous electrolyte^15^. Limitation of the substrate (H_2_O and OH^−^) in nonaqueous media permits the accumulation of active intermediate species, which allows the use of various in-situ spectroscopic measurements (infrared, UV-Vis, Raman, luminescence, and Mössbauer spectroelectrochemistry). They suggested that the O–O bond formed through the internal redox rearrangement mechanism of cis-dioxo-Fe(VI) to Fe(IV)-peroxide intermediate species.

Photosystems in nature adopt a Mn_4_Ca cluster to oxidize water into oxygen with high efficiency^16–18^. Interestingly, the geometric structure changes flexibly, and the spin state of the Mn_4_Ca cluster interconverts between low-spin (*S* = 1/2 with an open-cubane form) and high-spin (*S* = 5/2 with a closed-cubane form) forms during catalysis^19^. Inspired by this unique Mn_4_Ca cluster, various Mn-based heterogeneous catalysts have been developed^20–25^. However, the performance of man-made Mn-based heterogeneous catalysts has long suffered from the sluggish formation of Mn(III) species^26,27^. In general, the rigid lattice in Mn-based bulk inorganic crystals suppresses the generation of energetically favorable Jahn-Teller-distorted Mn(III) species, leading to slow kinetics of charge accumulation^27,28^. For this reason, considerable research effort has been dedicated to the generation and stabilization of Mn(III) intermediates. Previously, we demonstrated that Mn(III) species can be stably generated on the surface of manganese oxide NPs via a proton-coupled electron transfer pathway in a neutral phosphate electrolyte^29^ and further suggested that the rate-determining step involves Mn(IV)-oxo species rather than Mn(III) based on the in-situ/ex-situ spectroscopy analysis^30^.

Here we show that the engineering of the local distortions via Ni atom substitution in Mn_3_O_4_ NPs manipulates the spin state of the reaction intermediates during water oxidation reaction. Using a combination of experiment and computation, we explore the effect of Ni atom substitution on the intermediate species of nanoscale manganese oxide NPs. We find that the Ni substitution enables the compression of surface Mn octahedron, resulting in low-spin Mn(IV)-oxo intermediate species during the water oxidation reaction.

## Results and discussion

### Electrode preparation and electrochemical characterization

**Fig. 1:**
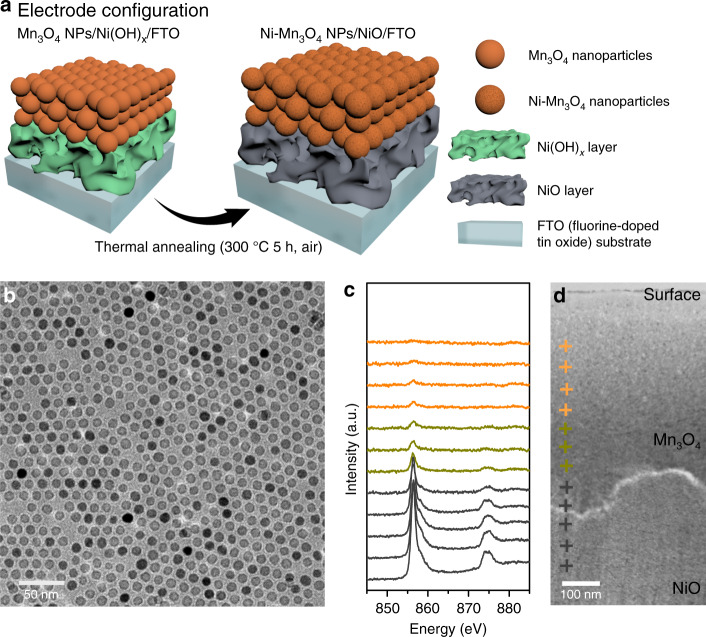
Electrode material characterization. **a** Schematic illustration of the electrode configuration of the nickel-decorated Mn_3_O_4_ NPs (Ni–Mn_3_O_4_ NPs). **b** TEM image of the monodispersed 10-nm Mn_3_O_4_ NPs. **c** HR-TEM EELS Ni L-edge spectra from the bottom (black) to the surface (orange) of Ni–Mn_3_O_4_NPs/NiO. The spot size for the EELS spectra is 10 nm. **d** Cross-sectional HR-TEM image of Ni–Mn_3_O_4_NPs/NiO. Each cross symbol corresponds to the EELS spectra in **c**.

Ni atom substitution in Mn_3_O_4_ NPs (Ni–Mn_3_O_4_ NPs) was accomplished through thermal annealing after nano-junction formation between the Ni source and Mn_3_O_4_ NPs. To create a nano-junction, a vertical-type Mn_3_O_4_ NPs/Ni(OH)_x_ layered structure electrode is designed (Fig. 1a). The detailed sample preparation of the Ni–Mn_3_O_4_ NPs/NiO layered structure is as follows. First, an amorphous nickel hydroxide layer was electrodeposited on a fluorine doped tin oxide (FTO) glass electrode. Monodisperse sub-10-nm Mn_3_O_4_ NPs, synthesized by thermal decomposition method (Fig. 1b), were then spin-coated onto the nickel hydroxide films. Calcination of the resulting electrode at 300 °C in air for 5 h yielded the Ni–Mn_3_O_4_ NPs/NiO/FTO electrode configuration (Fig. 1a). Approximately 400–500-nm-thick Ni–Mn_3_O_4_ NPs film was formed (Fig. 1d). High-resolution transmission electron microscopy (HR-TEM) analysis confirmed that the Mn_3_O_4_ NPs were not altered with respect to size and shape by the calcination treatment. Electron energy loss spectroscopy (EELS) line scans were used to examine the composition of the synthesized catalysts and revealed that the nickel species was uniformly distributed within the assembled NPs from the bottom interface between the Mn_3_O_4_ NPs layer and the NiO layer to the top surface of the electrode (Fig. 1c).

**Fig. 2:**
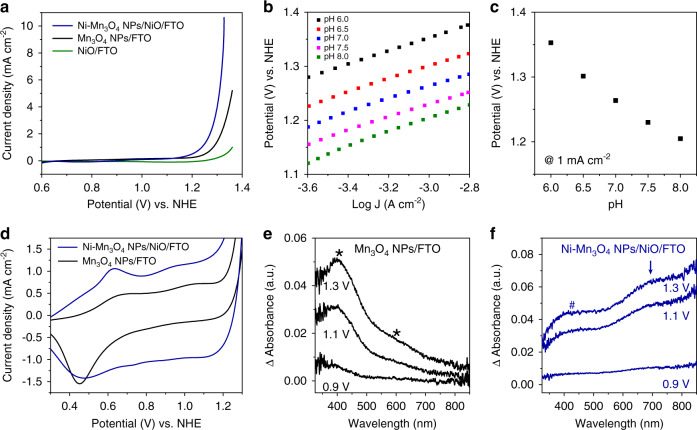
Electrochemical kinetic analysis and in-situ UV-Vis spectroscopic analysis. **a** Polarization-corrected CV curves for NiO, Mn_3_O_4_ NPs, and Ni–Mn_3_O_4_ NPs/NiO in 500 mM phosphate buffer (pH 7). **b** Tafel plot of Ni–Mn_3_O_4_ NPs/NiO at various pH. **c** pH dependency of Ni–Mn_3_O_4_ NPs/NiO under neutral conditions. **d** CV curves for Ni–Mn_3_O_4_ NPs/NiO and Mn_3_O_4_ NPs in 500 mM phosphate buffer (pH 7). Two distinct oxidation peaks were observed at ~0.6 and 1.0 V. According to a previous study, the first wave is attributable to the transition from Mn(II)-H_2_O to Mn(III)-OH. **e**, **f** In-situ UV-Vis spectroscopic analysis of Mn_3_O_4_ NPs and Ni–Mn_3_O_4_ NPs/NiO at 0.9, 1.1, and 1.3 V. Each differential spectrum was obtained by subtraction of the corresponding spectrum obtained at 0.8 V. Marked peak positions: * and # correspond to high-spin and low-spin Mn(IV), respectively.

The electrochemical properties of Ni–Mn_3_O_4_ NPs/NiO were characterized in 500 mM phosphate electrolyte (pH 7). Cyclic voltammetry (CV) curves were obtained and corrected by polarization to minimize the contribution of non-faradaic current to oxygen evolution reaction (OER) performance (Fig. 2a). Compared to the reference materials (Mn_3_O_4_ NPs and NiO), Ni–Mn_3_O_4_ NPs/NiO shows enhanced OER activity and an overpotential of 420 mV to reach 1 mA cm^−2^. Ni–Mn_3_O_4_ NPs/NiO sample exhibits a higher turnover frequency compared to Mn_3_O_4_ NPs sample, which is calculated by using the measured electrochemically active surface area (Supplementary Fig. 1 and Supplementary Table 1). The measured OER activity for Ni–Mn_3_O_4_ NPs/NiO and that for previously reported materials are summarized in Supplementary Fig. 2. The electrochemical kinetic and redox behaviors of Ni–Mn_3_O_4_ NPs/NiO upon water oxidation were further investigated in detail. As shown in Fig. 2b, c, the Tafel slopes and pH dependency were 70 mV dec^−1^ and –78 mV pH^−1^, respectively, in neutral phosphate electrolyte. The transfer coefficient was derived as ~1 from the Tafel slope, which indicates that the rate-determining process is a chemical step, preceded by a one-electron pre-equilibrium step^30,31^. The pH dependency indicates that log(j) has a first-order dependence on pH and that the current density has an inverse first-order dependence on proton activity. Thus, concerted one-proton and one-electron transfer reactions occur as a pre-equilibrium step, followed by the chemical rate-determining process^31^. Similar electrokinetic behavior was observed in manganese oxide NPs^30^.

In addition, two distinct oxidation peaks were observed at 0.6 and 1.0 V (Fig. 2d). According to a previous study^30^, the oxidation peak at 0.6 V corresponds to the transition from Mn(II)-H_2_O to Mn(III)-OH. We thus speculated that high-valent Mn(IV) species are generated as reaction intermediates in the Ni–Mn_3_O_4_ NPs/NiO system during the water oxidation reaction. In-situ X-ray absorption near edge structure (XANES) analysis (Supplementary Fig. 3) further supported the observed Mn redox behavior, as the average Mn valence in Ni–Mn_3_O_4_ NPs/NiO increased from 2.83 to 3.45 with increasing applied potential, whereas the valence state of Ni is only slightly increased.

### In-situ and ex-situ spectroscopic analysis

To capture the generated intermediate Mn species on the NPs surface, potential-dependent in-situ UV-Vis spectra for the Mn_3_O_4_ NPs and Ni–Mn_3_O_4_ NPs/NiO electrodes were monitored at pH 7.0 (Fig. 2e, f). Absorbance spectra at each potential were obtained by subtracting the spectra obtained at 0.8 V, i.e., before the onset potential for water oxidation. In accordance with the results of previous studies, ligand-to-metal charge transfer (LMCT, ~400 nm) and *d*-*d* transition (~600 nm) bands, which are characteristic features of high-spin Mn(IV)-oxo species, were detected in the spectra of Mn_3_O_4_ NPs (Fig. 2e)^30^. Clearly, Ni–Mn_3_O_4_ NPs/NiO catalyst exhibits a shifted peak at 420 nm (denoted as # in Fig. 2f) and an intense absorption change between 550 and 800 nm region (denoted as an arrow in Fig. 2f). The shifted peak at 420 nm is originated from new intermediate species from Ni–Mn_3_O_4_ NPs layer. The calculation results, which will be discussed later, support that the origin of the red-shifted peak at 420 nm is a π-type LMCT band of the new intermediate species. It is turned out that the broad peak between 550 and 800 nm is due to the overlapping between the new intermediate’s another LMCT band and the absorption of nickel oxyhydroxide formed by the oxidation of underlying NiO layer^32,33^.

**Fig. 3:**
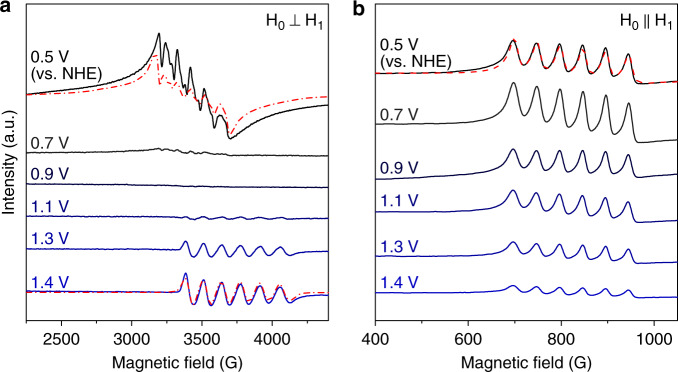
Measured and simulated potential-dependent EPR spectra. **a**, **b** Potential-dependent X-band CW-EPR spectra of Ni–Mn_3_O_4_ NPs/NiO in perpendicular (**a**) and parallel (**b**) modes. The dashed red lines are spectral simulations that are well matched with the experimental curves.

Potential-dependent ex-situ electron paramagnetic resonance (EPR) measurements were conducted by freeze-quenching the samples to understand the electronic structure of the intermediates. The catalysts on the FTO substrates were collected immediately after electrolysis and subsequently quenched in liquid nitrogen. Dual-mode (perpendicular and parallel mode) continuous-wave EPR (CW-EPR) spectra of Ni–Mn_3_O_4_ NPs/NiO at various anodic potentials are shown in Fig. 3 and Supplementary Fig. 4. The initial state of Ni–Mn_3_O_4_ NPs/NiO exhibited characteristic features of high-spin Mn(II)/Mn(III). A characteristic high-spin Mn(II) species (*S* = 5/2) with six-line hyperfine splitting at *g* ~ 2 was detected in perpendicular mode. A Mn signal measured in parallel mode was attributed to typical high-spin Mn(III) (*S* = 2) species. As the applied potential increased to 0.7 V, the intensity of the Mn(II) signals gradually diminished, whereas the intensity of the Mn(III) signals increased. When the applied potential further increased, the Mn(II) signal disappeared and the intensity of the Mn(III) signal gradually decreased, which indicates the formation of higher-valent Mn species (Supplementary Fig. 5).

At applied potentials >1.1 V (vs. NHE), unique spectral behavior for Ni–Mn_3_O_4_ NPs/NiO is observed. As shown in Fig. 3a and Supplementary Fig. 4, the perpendicular-mode EPR spectrum of potential-applied Ni–Mn_3_O_4_ NPs/NiO exhibits a new six-line signal centered at *g* ~ 1.83, which was not found in the spectrum of non-treated Mn_3_O_4_ NPs. This signal continuously evolved as the anodic potential increased. To confirm the importance of uniform incorporation of Ni into Mn_3_O_4_ NPs to obtain new EPR signal, several types of electrodes were fabricated, and the presence of a new EPR signal was investigated (Supplementary Fig. 6). No signal was observed near *g* ~ 1.83 during the OER in the case of the electrodes whose Ni bottom layer was prepared by the sputtering method (Ni and NiO). Ni atoms might not diffuse sufficiently into the Mn_3_O_4_ NPs layer during thermal annealing (300 °C 5 h) because the sputtered Ni bottom layers (Ni and NiO) have high crystallinity (Supplementary Fig. 7). However, although the intensity of the signal was different, the same signal was also observed in the spectrum of the simple mixed type (Mn_3_O_4_ NPs and NiO NPs) electrode, which implies the possibility for generalization of our design rule (Supplementary Figs. 8 and 9). To our knowledge, a Mn signal centered at *g* ~ 1.83 has not been reported to date. Based on the results of previous EPR studies^34,35^, we first excluded the possibility that this signal was due to Mn(V) or high-spin Mn(IV) species. There is no report on the separation and detailed spectroscopic characterization of low-spin Mn(IV)-oxo, even in homogeneous Mn complexes, although low-spin Mn(IV) nitride and possible detection of a low-spin Mn(IV)-oxo generated from a Mn(V)-oxo porphyrin complex were reported^36,37^. Reported low-spin Mn(IV) nitride complex has tetrahedral geometry and exhibit rhombic EPR signal (***g*** = [2.35, 1.973, 1.965], |***A***| = [300, 74, 202] MHz)^36^. We consider the possibility that the newly detected EPR signal (*g* ~ 1.83) can be attributed to a low-spin Mn(IV)-oxo species in octahedral geometry (*S* = 1/2).

**Fig. 4:**
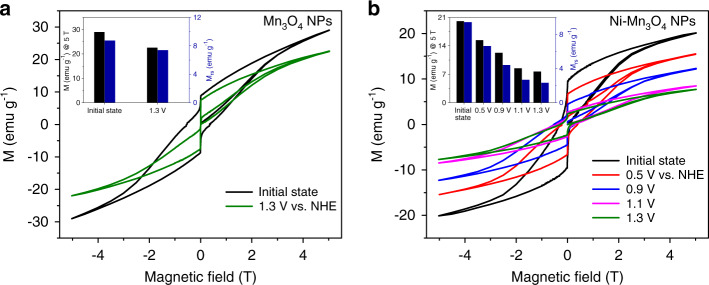
Potential-dependent magnetization measurements using a SQUID. **a**, **b** Potential-dependent magnetic hysteresis curves for Mn_3_O_4_ NPs **a**, and Ni–Mn_3_O_4_ NPs/NiO **b**, measured at 5 K. Compared to Mn_3_O_4_ NPs, Ni–Mn_3_O_4_ NPs/NiO shows significantly diminished magnetization under the applied anodic potential. The black and blue histograms in inset figures show the magnetization values at 5 T and 0 T (*M*_rs_), respectively.

Direct evidence for low-spin Mn(IV) is also obtained using potential-dependent superconducting quantum interference device (SQUID) analysis. Since the saturated magnetization value (*M*_s_) is directly proportional to the total spin number, the spin number can be estimated by measurement of the magnetization value (*M*) at 5 T and the saturation remanence (*M*_rs_). Potential-dependent M-T curves for pristine Mn_3_O_4_ NPs and Ni–Mn_3_O_4_ NPs were recorded at 5 K. As shown in Fig. 4, in either case, the *M* value at 5 T (*M*_5T_) and the *M*_rs_ value progressively decrease due to the formation of higher-valent Mn(IV). The *M* value at 5 T for Mn_3_O_4_ decreased from 27 erg g^−1^ to 19 erg g^−1^ at an applied potential of 1.3 V, and a more significant decrease in the magnetization values was observed for Ni–Mn_3_O_4_ NPs. In addition, *M*_rs_ decreased by approximately one-third at 1.3 V compared to the initial value, presumably due to the formation of low-spin Mn(IV).

The detailed electron configuration of low-spin Mn(IV) is confirmed by EPR. The exact EPR parameters of the signal were extracted from the simulation of the EPR spectrum. The EPR simulation yielded *g*-values of ***g*** = [1.84, 1.84, 1.82] and a hyperfine tensor of |***A***| = [121, 121, 132] G, which agree well with the experimental spectra. The simulated spectrum is fairly sensitive to changes in the simulated parameters. Simulated spectra with the various parameters are displayed in Supplementary Fig. 10, confirming the accuracy of the fitting result. The *g*-values of low-spin Mn(IV) (*S* = 1/2, *d*^3^) exhibit a large deviation from the free-electron value *g*_e_. These abnormal *g*-values may be affected by the unique geometric and electronic structure of the intermediate species.

**Fig. 5:**
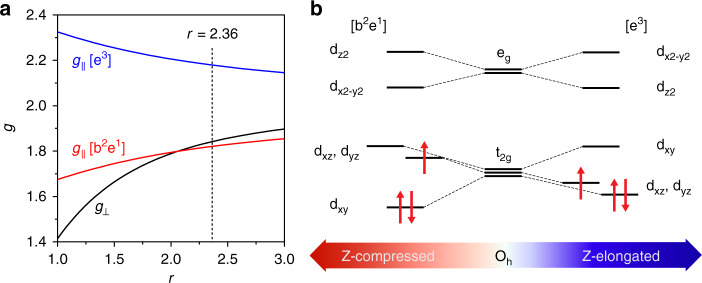
Evaluation of *g*-values and corresponding *d*-orbital configurations of the Mn(IV) species. **a** Plot of *g*-values for the [b^2^e^1^] and [e^3^] configurations as a function of *r***. b** Qualitative *d*-orbital splitting pattern for tetragonally compressed ([b^2^e^1^] configuration) and elongated ([e^3^] configuration) low-spin octahedral Mn(IV) centers.

Although only a few studies of low-spin *d*^3^ systems have been reported, one can consider the pseudo Jahn-Teller (PJT) effect of the Mn(IV) species where spin–orbit coupling competes with the interactions that split the orbital degeneracy, thus lowering the molecular symmetry to C_4v_ symmetry^38^. In fact, the low-spin Mn(IV) in the Ni–Mn_3_O_4_ NPs/NiO intermediate has two possible *d*-orbital configurations (^2^E states; [e^3^] and [b^2^e^1^]) (Fig. 5). The *g*-values can be described in terms of the ratio of the sum of the vibronic coupling energy at the equilibrium distortion (*V*_vib_) and the solvent-dependent environmental energies affecting the orbital splitting (*V*_L_) to the spin–orbit coupling constant (*λ*)^39^, through the fictitious angle *2θ*:

1 $$r = \frac{{2\left( {V_{{\mathrm{vib}}} + V_{\mathrm{L}}} \right)}}{\lambda } = {\mathrm{tan}}2\theta$$

2 $${g}_ \bot = 2\,{\mathrm{sin}}2\theta$$

3 $$\left[ {{\mathrm{e}}^3} \right]{g}_\parallel = 2\left( {1 + k\,{\mathrm{cos}}2\theta } \right)$$

4 $$\left[ {{\mathrm{b}}^2{\mathrm{e}}^1} \right]{g}_\parallel = 2\left( {1 - k\,{\mathrm{cos}}2\theta } \right)$$

where *k* is the *d*-electron delocalization factor (0 (fully delocalized) ≤ *k* ≤ 1 (free metal ion))^40–43^. As shown in Fig. 5a, Eqs. (3) and (4) indicate that the [b^2^e^1^] configuration corresponds to *g*-values of [*g*_∥_ ≤ *g*_⊥_ ≤ 2], whereas the [e^3^] configuration corresponds to *g*-values of [*g*_⊥_ ≤ 2 ≤ *g*_∥_]. Additionally, the [b^2^e^1^] configuration represents the tetragonally compressed octahedral geometry of the Mn(IV) center while the [e^3^] configuration represents the tetragonally elongated octahedral geometry (Fig. 5b). From the simulated values of *g*_∥_ = 1.82 and *g*_⊥_ = 1.84, *k* is estimated to be 0.23 and these *g*-values for the low-spin Mn(IV) species indicate the [b^2^e^1^] configuration, which represents a tetragonally compressed octahedral geometry.

In addition, the PJT splitting energy (*E*_PJT_), which indicates the stabilization resulted from the breaking of the orbital degeneracy, can be estimated from the *r* value and *g*-values as *E*_PJT_ = *λ*[1 + tan^2^2*θ*]^1/2^ = 1000 cm^–1^ by using Eqs. (1)–(3)^40,42^. This PJT splitting is characteristic of the distorted *C*_4v_ symmetry of the Mn(IV) species, where the degeneracy of the *d*_xz_ and *d*_yz_ orbitals is broken^38^. Thus, the estimation of this *d*-orbital configuration provides valuable information about the local geometry and the electronic structure of the Mn(IV) center. We conceive that the atomic origin of the (slightly) broken degeneracy of the *d*_xz_ and *d*_yz_ orbitals is due to the disrupted rhombicity of the equatorial ligand field near the Mn(IV) center.

**Fig. 6:**
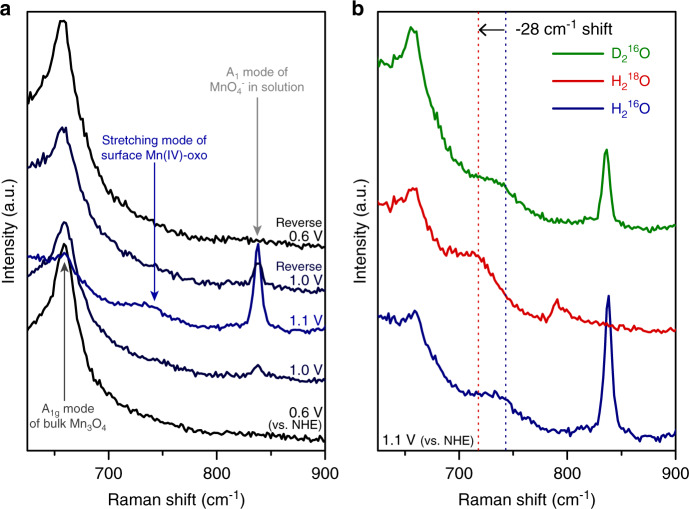
In-situ Raman analysis. **a** Potential-dependent in-situ Raman spectra of Ni–Mn_3_O_4_ NPs/NiO/FTO measured by increasing the applied potential from 0.6 V (vs. NHE) to the above of onset potential (1.1 V vs. NHE) and then decreased to the 0.6 V (vs. NHE). **b** In-situ Raman spectra during the water oxidation (1.1 V vs. NHE) under various electrolytes. The observed broad Raman peak found in H_2_^16^O electrolyte (at 744 cm^−1^) is red-shifted to 716 cm^−1^ in ^18^O labeled electrolyte, whereas there is no peak shift in the D_2_^16^O electrolyte.

Furthermore, potential-dependent in-situ Raman spectroscopy analysis was conducted to get direct evidence for oxo species (Fig. 6 and Supplementary Fig. 11). The sharp and intense Raman peak at 660 cm^−1^, corresponding to the *A*_1g_ vibrational mode of spinel crystal structure^44^, is visible at the open circuit potential (Fig. 6a). Note that the Raman peak of soluble MnO_4_^−^ is observed at 837 cm^−1^ before the onset potential (1.0 V vs. NHE)^45^. When the anodic potential is above the onset potential for water oxidation reaction (1.1 V vs. NHE), a broad Raman peak near 744 cm^−1^ is developed. This broad Raman peak reversibly disappears when the applied potential is below the onset potential, which indicates that the origin of this Raman peak is from the active intermediate species. From the previously studied molecular complexes, reported Raman peak positions for stretching vibrational mode of Mn(IV)-oxo species are between 707 and 803 cm^−1^ (refs. ^46–48^), while those of Mn(V)-oxo species are between 957 and 997 cm^−1^ (refs. ^49–51^). Therefore, it is reasonable to conclude that the broad Raman peak is attributed to the stretching vibrational mode of Mn(IV)-oxo species.

To further confirm the nature of the Mn(IV)-oxo species, in-situ Raman spectra are also obtained in the H_2_^18^O and D_2_^16^O electrolyte (Fig. 6b). The broad Raman peak at 744 cm^−1^ is red-shifted to 716 cm^−1^ in the H_2_^18^O electrolyte. The oxygen isotope shift of 28 cm^−1^ with ^18^O substitution is well matched with the expected value of 33 cm^−1^ from a diatomic oscillator calculation of Mn(IV)-oxo species. Besides, the positions of Raman peaks remain unchanged in the D_2_^16^O electrolyte compared to the spectra obtained in the H_2_^16^O, which means that the captured intermediate species do not include hydrogen atom. Labeling experimental results clearly prove that the broad Raman band at 744 cm^−1^ can be assigned to the stretching vibrational mode of Mn(IV)-oxo species.

### DFT calculations

**Fig. 7:**
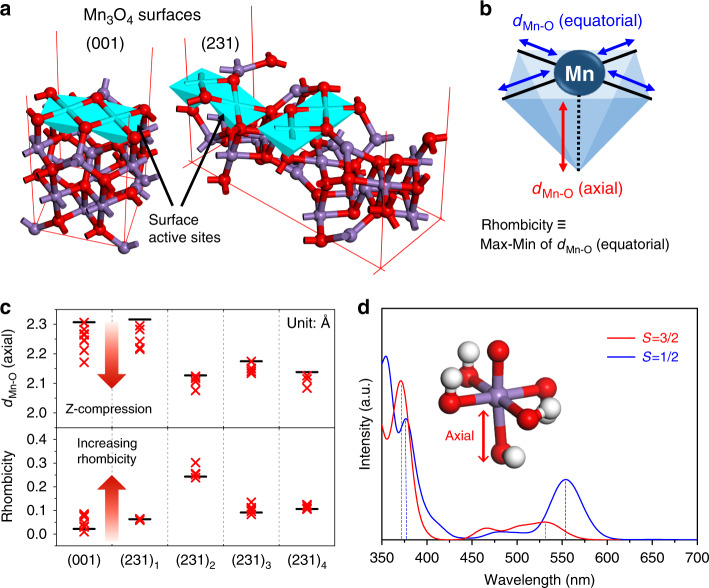
DFT calculations to evaluate the effect of Ni substitution. **a** Atomic structures of Mn_3_O_4_ (001) and (231) surfaces used in periodic DFT calculations. **b** Schematic illustration of the surface Mn active site with the definition of rhombicity. **c** The effect of Ni substitution on the axial Mn–O distance and rhombicity of equatorial Mn–O distances of the Mn_3_O_4_ (001) and (231) surface active sites. Here, rhombicity is quantified as the difference between the shortest and longest equatorial Mn–O bonds. **d** Calculated UV-Vis spectra of the high-spin and low-spin Mn(IV) site cluster models (inset) showing redshifts.

Using DFT, we attempted to elucidate the origin of the low-spin Mn(IV) species and identify the spectroscopic features of Mn(IV) species that can be compared with our experimental results. From periodic DFT calculations with a Hubbard-type correction (DFT+*U*) method, we optimized the structure of the Ni–Mn_3_O_4_ NPs (001) and (231) surfaces that are representative low- and high-index surfaces found in our NPs (Fig. 7a and Supplementary Fig. 12). On such facets, we generally found that Ni substitution into the Mn_3_O_4_ substantially reduced the axial Mn–O distance (a compression of *d*_Mn–O_(axial)), and disrupted the rhombicity (an increase in Max-Min *d*_Mn–O_(equatorial)) of the truncated MnO_6_ octahedron at the surface (Fig. 7b, c, Supplementary Fig. 13, and Supplementary Table 2). This finding suggests that the Ni substitution into the Mn–O network around the surface active site increases the axial crystal field and slightly break the degeneracy of the *d*_xz_ and *d*_yz_ energy levels (Supplementary Fig. 14). Thus, the axial crystal field becomes stronger after Ni substitution due to the shortened *d*_Mn–O_(axial); therefore, the $d_{z^2}$ and *d*_xz/yz_ orbitals become destabilized, yielding an increased energy gap between *d*_xz/yz_ and *d*_xy_. This increased gap provides an energetic driving force for the high-spin to low-spin transition, and yields an electronic configuration of [b^2^e^1^] as shown in Fig. 5.

To mimic the ligand field effect on the Mn(IV) center imposed by the Mn_3_O_4_ lattice, we built a cluster model with a Mn(IV) center coordinated by five OH^–^ ligands, the oxygen positions of which were fixed at the same as in the periodic DFT-optimized structure of Ni-doped Mn_3_O_4_ (inset of Fig. 7d and Supplementary Fig. 15). Then, an additional oxygen atom was adsorbed to the Mn center to form the Mn(IV)=O bond. We note that the atomic positions of H and the adsorbate O were fully relaxed using a non-periodic DFT calculation including the spin-orbit effect. For comparison, we also performed a non-periodic DFT calculation for the high-spin Mn(IV)-oxo species (*S* = 3/2) using the same cluster model.

The non-periodic DFT calculation for the low-spin Mn(IV)-oxo species resulted in a large and fairly isotropic value of hyperfine coupling constants |***A***| = [158, 143, 135] G, which is in good agreement with the experimental values of |***A***| = [121, 121, 132] G. Notably, the DFT calculation for the high-spin Mn(IV)-oxo species yielded a much smaller value of the hyperfine tensor |***A***| = [44, 43, 49] G, which was intuitively anticipated for a high-valent metal center in a high-spin state. Thus, the experimentally observed large value of |***A***| supports the existence of a low-spin Mn(IV)-oxo species. Furthermore, the isotropic |***A***| values of the low-spin Mn(IV)-oxo species can be explained by the fairly isotropic shape of the spin density (Supplementary Fig. 16), which originated from the electronic configuration of [b^2^e^1^], where the singly occupied molecular orbital (SOMO) has *d*_xz_/*d*_yz_ character.

Using the same cluster models, we further calculated the UV-Vis spectrum of Mn(IV)-oxo species using time-dependent DFT (Fig. 7d and Supplementary Fig. 17). As shown in Fig. 7d, the π-type LMCT band for the high-spin Mn(IV)-oxo is found at 370.5 nm, while that for the low-spin Mn(IV)-oxo is found to be red-shifted to 375.2 nm, which agrees with our experimentally measured UV-Vis spectrum. In addition, the high-spin Mn(IV)-oxo exhibits a *d*–*d* transition band at 534.5 nm, while the low-spin Mn(IV)-oxo exhibits another LMCT band at 554.1 nm due to the broken degeneracy of *d*_xz_ and *d*_yz_ orbital. Therefore, low-spin Mn(IV)-oxo shows a relatively intense absorption peak at 554.1 nm compared to high-spin Mn(IV)-oxo.

### Conclusion

In summary, a new intermediate species was experimentally captured by in-situ UV-Vis, potential-dependent freeze-quenching EPR, and in-situ Raman spectroscopy during the electrochemical water oxidation reaction of Ni–Mn_3_O_4_ NPs. Based on the results of the Raman experiment, EPR simulation and DFT calculation, we assigned this intermediate species as a low-spin Mn(IV)-oxo with a [b^2^e^1^] electronic configuration. This unique configuration was a result of the axial compression of an octahedral Mn(IV)-oxo species induced by Ni substitution, where the energy gap between the *d*_xz/yz_ and *d*_xy_ orbitals increased. In particular, the characteristic features of spectroscopic data, namely, (1) large and isotropic EPR hyperfine coupling constants |***A***|, (2) an electronic configuration of [b^2^e^1^] derived from EPR *g*-values, and (3) redshifts in the UV-Vis spectrum, are fully supported by the DFT calculation of a Mn(IV)-oxo complex with *S* = 1/2. We propose that such good agreement between theory and experiment corroborates the formation of the low-spin Mn(IV)-oxo species. Our findings provide new insight into the heterogeneous electrocatalyst design for intermediate characterization and manipulation. Furthermore, we expect that the findings from this work may extend into other fields beyond water oxidation reactions, such as fuel cells, CO_2_ reduction, biomass conversion, and enzyme mimetic reactions, which utilize transition metal-based heterogeneous catalysts.

## Methods

### Materials

Mn(CH_3_COO)_3_-2H_2_O (99%), Ni(CH_3_COO)_2_-4H_2_O (99%), 1-octadecene (90%), myristic acid (99%), decanol (98%), MnO (99.99%), Mn_3_O_4_ (97%), Mn_2_O_3_ (99.9%), NaH_2_PO_4_-H_2_O (99.0%, ACS reagent), and Na_2_HPO_4_-7H_2_O (98.0–102.0%, ACS reagent) were purchased from Sigma Aldrich. Ni(NO_3_)_2_–6H_2_O (99.9985%), MnO_2_ (99.9%), and Pt foil (99.997%) were purchased from Alfa Aesar. Acetone (99.5%), toluene (99.5%), hexane (95%), cyclohexane (99.5%), and ethanol (99.5%) were purchased from Daejung Chemicals. All chemicals used as received without further purification. Fluorine doped tin oxide coated glass (FTO) with the surface resistivity of 8 Ω sq^–1^ was obtained as 1.0 cm × 1.5 cm pre-cut glass pieces.

### Electrode preparation

Synthesis of metal oxide nanoparticles (Mn_3_O_4_ NPs and NiO NPs).

Manganese oxide and nickel oxide NPs were synthesized by thermal decomposition and hot-injection methods. To prepare sub-10-nm monodispersed metal oxide NPs, separate reaction pots were used. One pot contained 1 mmol of M(ac)_3_ (M = Mn and Ni) and 2 mmol of myristic acid dissolved in 20 mL of octadecene, and the other pot contained 3 mmol of decanol dissolved in 1 mL of octadecene. The two separate mixtures were degassed at 110 °C for 1 h with vigorous stirring, and, then, the carboxylate mixture was heated above 295 °C in an argon atmosphere. When the temperature reached 295 °C, the decanol solution was injected rapidly into the carboxylate solution to induce burst nucleation. The reaction mixture was incubated at 295 °C for 1 h followed by cooling to room temperature. A 1:1:1 ratio of the solution, acetone and toluene was mixed and centrifuged to remove organic residues from the solution. After repeating the purification step, the metal oxide NPs were re-dispersed in nonpolar solvents, such as hexane and cyclohexane.

### Mn_3_O_4_ NPs + NiO NPs/FTO configuration

First, the FTO substrate was sequentially washed by sonication with acetone, ethanol, and deionized water. Then, synthesized Mn_3_O_4_ NPs and NiO NPs were mixed and dispersed in hexane. A film was prepared by spin coating the hexane solution onto a FTO substrate. Further heating at 300 °C for 5 h in an air atmosphere was performed to enhance adhesion to the substrate along with the Ni substitution into the Mn_3_O_4_ NPs lattice.

### Ni–Mn_3_O_4_ NPs/NiO/FTO configuration

Substrate materials and amorphous nickel oxide films were fabricated by electrodeposition method. To determine the deposition conditions, we first measured CV curves in a nickel nitrate solution (90 mM aqueous solution). A nickel redox peak was observed at ~–1.0 V vs Ag/AgCl. Based on the results, the electrolysis potential was set to –1.1 V. Since the charge passed during the depositions is proportional to the deposition amount, we deposited amorphous nickel hydroxide film with 300 mC  cm^–2^. Then, 10 nm Mn_3_O_4_ NPs were spin-coated onto Ni(OH)_X_ films. Subsequently, further heating at 300 °C for 5 h under aerobic conditions was performed to enhance adhesion to the substrate along with Ni substitution into the Mn_3_O_4_ NPs lattice.

### Materials characterization

The morphology of Ni–Mn_3_O_4_ NPs/NiO was characterized using a high-resolution scanning electron microscope (Supra 55VP, Carl Zeiss, Germany). Pt coating was carried out using a Pt sputter coater (BAL-TEC/SCD 005). Images were obtained using an acceleration voltage of 2 kV, and energy-dispersive X-ray (EDX) spectra were obtained at 15 kV. The sample positions coincided with the illuminated area. HR-TEM images and selected area electron diffraction (SAED) patterns were obtained using a high-resolution transmission electron microscope (JEM-2100F, JEOL, Japan) with an acceleration voltage of 300 kV. The TEM samples were collected from FTO glass and dispersed in ethanol by sonication for ~1 min.

### Electrochemical analysis

All electrochemical experiments were conducted using a three-electrode electrochemical cell system. A BASi Ag/AgCl/3 M NaCl reference electrode and Pt foil (2 cm × 2 cm × 0.1 mm, 99.997% purity, Alfa Aesar) were used as the reference and counter electrodes, respectively. Electrochemical tests were conducted at ambient temperature (21 ± 1 °C) using a potentiostat system (CHI 760E, CH Instruments, Inc.). The electrode potential was converted to the NHE scale using the following equation: E(NHE) = E(Ag/AgCl) + 0.210 V. Additionally, overpotential values were calculated from the difference between the iR corrected potential (*V* = *V*_applied_ – iR) and the thermodynamic point for water oxidation at a specified pH. The electrolyte was 500 mM phosphate buffer at pH 7. The working electrode was cycled to obtain a CV curve without pauses at a scan rate of 10 mV s^–1^. Prior to conducting the electrochemical experiments, the solution resistance was measured in an electrolysis bath. All of the data were iR-compensated. The Tafel slope was calculated from the steady-state current density that is measured by bulk electrolysis with various applied potentials. The pH dependence was measured by chronopotentiometric analysis at a constant current density (1 mA cm^–2^). Then, the pH dependency on the current density was calculated with the following formula (Eq. (1)) combined with the Tafel slope results:

1 $$\left( {\frac{{\partial E}}{{\partial {\mathrm{pH}}}}} \right)_j = - \left( {\frac{{\partial E}}{{\partial {\mathrm{log}}j}}} \right)_{{\mathrm{pH}}}\left( {\frac{{\partial {\mathrm{log}}j}}{{\partial {\mathrm{pH}}}}} \right)_E$$

According to the formula, we can determine the dependence of log(*j*) on pH.

### EPR study

All EPR measurements were carried out at the Korea Basic Science Institute (KBSI) in Seoul, Korea. The simulations of the EPR spectra were carried out by EasySpin^52^. CW X-band (9.6 GHz) EPR spectra were collected using a Bruker EMX plus 6/1 spectrometer equipped with an Oxford Instruments ESR900 liquid-He cryostat using an Oxford ITC 503 temperature controller. Low temperatures were achieved and controlled using an Oxford Instruments ESR900 liquid-He quartz cryostat with an Oxford Instruments ITC 503 temperature and gas flow controller. The experimental conditions were as follows: microwave frequencies of 9.64 GHz (perpendicular mode) and 9.40 GHz (parallel mode), a modulation amplitude of 10 G, a modulation frequency of 100 kHz, microwave powers of 0.94 mW (perpendicular mode) and 5.0 mW (parallel mode), and a temperature of 5.7 K. Five scans were combined for each spectrum. The bulk electrolysis was conducted using a potentiostat (CHI 600D CH Instruments, Inc.) at pH 7 in 500 mM phosphate buffer solution. Set potentials were applied to each sample for 15 min. After carrying out the bulk electrolysis, the samples were rinsed gently with deionized water and rapidly transferred to an EPR tube using a blade. The EPR tubes were then immediately frozen and stored at 77 K in liquid nitrogen.

### In-situ Raman study

All the in-situ Raman spectroscopy analyses were performed with a Raman microscope (LabRAM HR Evolution, Horiba) with a ×50 long working distance (LWD) visible objective lens. Raman spectra were collected with a 532-nm excitation laser source with a laser power of 1.6 mW. The individual Raman spectrum was obtained with an acquisition time of 300 s with 600 g mm^−1^ grating. To ensure the reliability of in-situ measurements, the Raman shift was calibrated using a silicon standard sample (520.6 cm^−1^) before the initial acquisition of Raman spectra. The in-situ measurements were implemented in a home-made electrochemical cell with a three-electrode configuration at room temperature. To eliminate the possible overlapping of the target Mn-oxo peak to the peaks of the phosphate electrolyte, here we utilized 1 M KHCO_3_ electrolyte (pH 8.2) for the in-situ Raman measurement. The Raman spectrum at each applied potential was obtained via bulk electrolysis using a potentiostat (CHI 600E CH Instruments, Inc.) and the acquisition was initiated after the current was attained to steady-state.

### Computational study

DFT calculations were carried out using the Vienna Ab Initio Software Package (VASP)^53,54^ to understand the characteristics of the Mn_3_O_4_ surface system with Ni substitution. All calculations were carried out using the Perdew-Burke-Ernzerhof (PBE) exchange-correlation functional^55^ and the electron-ion interaction was considered in the form used by the projector-augmented-wave (PAW) method^56^ with a plane wave cutoff energy of 400 eV. To adjust the electron correlation contribution for Mn and Ni 3*d* electrons, a PBE+*U* scheme was adopted with an effective *U* value of 5.0 eV. The Mn_3_O_4_ (001) and (231) surface models, with an additional 20 Å vacuum layer along the *z*-axis, were prepared using the optimized Mn_3_O_4_ bulk structure. Gamma-centered k-point grids of (6 × 6 × 1) and (5 × 2 × 1) were employed for the (001) and (231) surfaces, respectively. A dipole correction was applied along the *z*-direction to properly compensate for the artificial dipole interactions across the periodic boundary. Non-periodic DFT calculations for the cluster models were carried out using Orca 4.1^57^, to compute the EPR hyperfine coupling constant and the UV-Vis spectra. We used the Becke three-parameter functional (B3) combined with the correlation functional of Lee, Yang, and Parr (LYP)^58^, which is known as the proper choice for modeling spin crossover phenomena, and the ZORA-def2-TZVP SARC/J^59^ basis set functions. Relativistic spin-orbit coupling was included using a zeroth-order regular approximation (ZORA)^60^ for both the structural optimizations and the time-dependent DFT (TD-DFT) calculations. UV-Vis spectra were calculated using TD-DFT with the Tamm-Dancoff approximation^61^.

## Supplementary information

Supplementary Information

Peer Review File

## Data Availability

All data is available in the main text or the supplementary information.
