# Supplementary material for: Spectroscopic capture of a low-spin Mn(IV)-oxo species in Ni–Mn3O4 nanoparticles during water oxidation catalysis
Source: Nat Commun. 2020 Oct 16;11:5230. doi: 10.1038/s41467-020-19133-w (PMC7567882; doi:10.1038/s41467-020-19133-w)
Supplement: Supplementary file 1 — Supplementary Information [file 41467_2020_19133_MOESM1_ESM.pdf]

Supplementary Information

Spectroscopic capture of a low-spin Mn(IV)-oxo species in Ni-Mn_3O_4 nanoparticles during water oxidation catalysis

Authors: Sunghak Park†, Kyoungsuk Jin†, Hyung Kyu Lim, Jin Kim, Kang Hee Cho, Seungwoo Choi, Hongmin Seo, Moo Young Lee, Yoon Ho Lee, Sangmoon Yoon, Miyoung Kim, Hyungjun Kim*, Sun Hee Kim*, and Ki Tae Nam*

† These authors contributed equally to this work

*** To whom correspondence should be addressed:**

Hyungjun Kim, Ph.D. (linus16@kaist.ac.kr), Sun Hee Kim, Ph.D. (shkim7@kbsi.re.kr), and Ki Tae Nam, Ph.D. (nkitae@snu.ac.kr),

This file includes:

Supplementary Figure 1 to 17

Supplementary Table 1 and 2

Supplementary References

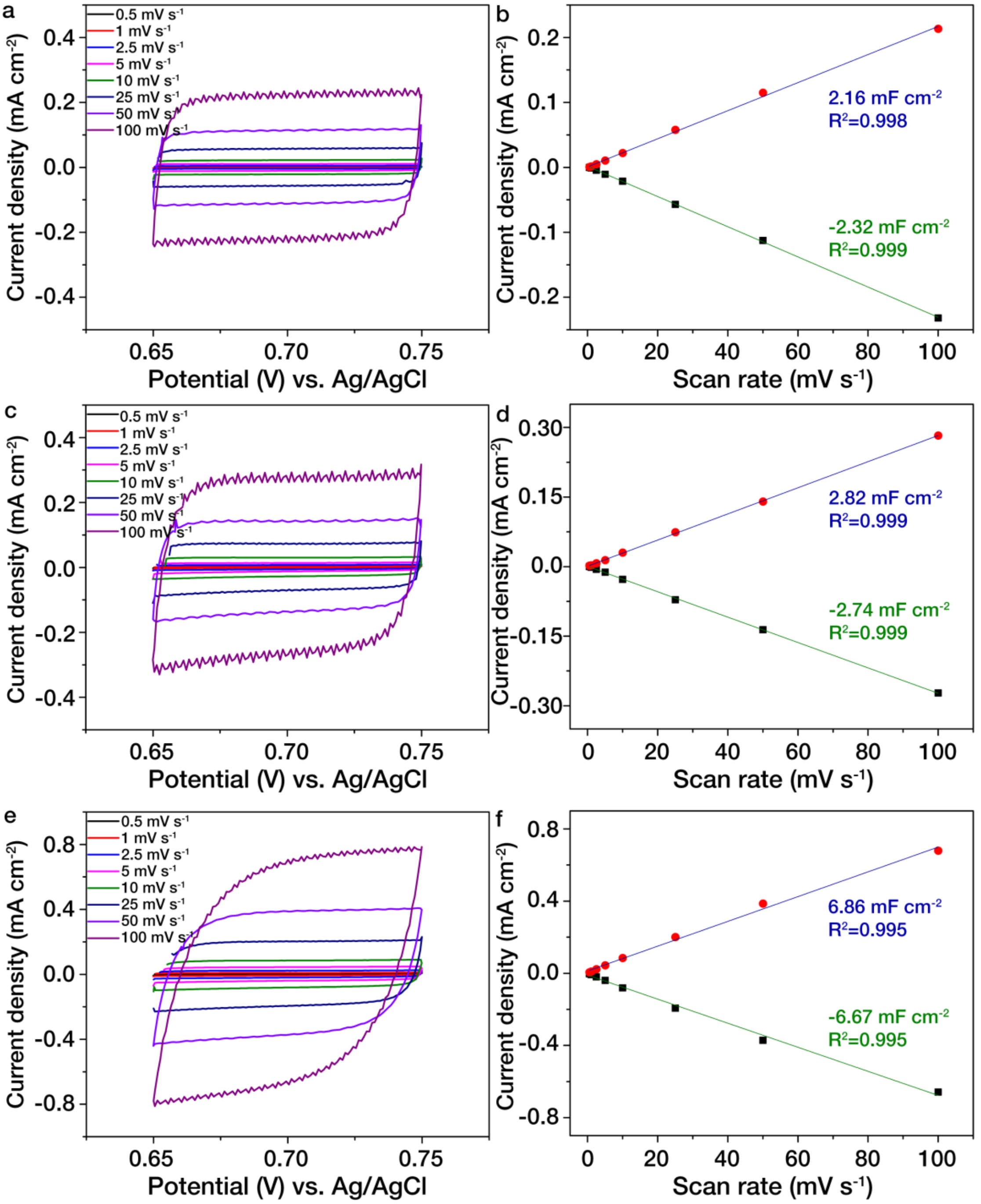

Supplementary Figure 1 | The electrochemically active surface area of prepared electrodes. Cyclic voltammograms of NiO/FTO (**a**), Mn_3O_4 NPs/FTO (**c**), Ni-Mn_3O_4 NPs/NiO/FTO (**e**) in 0.5 M phosphate buffer (pH 7). Plots of the anodic and cathodic current densities versus scan rates (**b**: NiO/FTO, **d**: Mn_3O_4 NPs/FTO, and **f**: Ni-Mn_3O_4 NPs/NiO/FTO). The double-layer capacitance is derived from the slopes of linear fitting results.

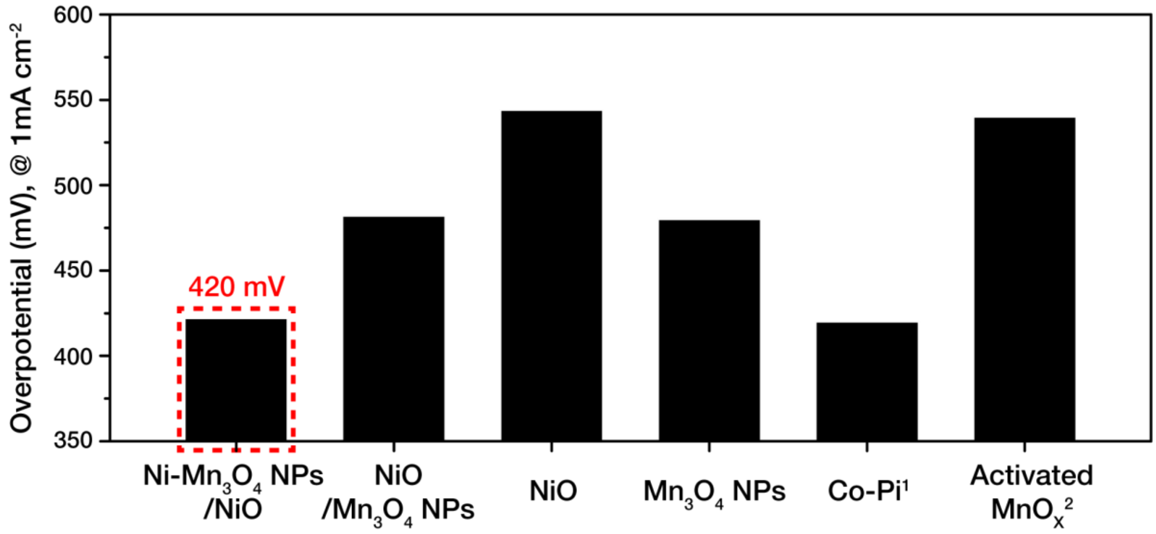

Supplementary Figure 2 | Overpotential values required to reach 1 mA cm^{-2} at neutral pH for various water oxidation catalysts. Synthesized Ni-Mn_3O_4 NPs/NiO catalyst exhibits enhanced catalytic activity in 500 mM phosphate electrolyte (pH 7) compared to previously reported catalysts.

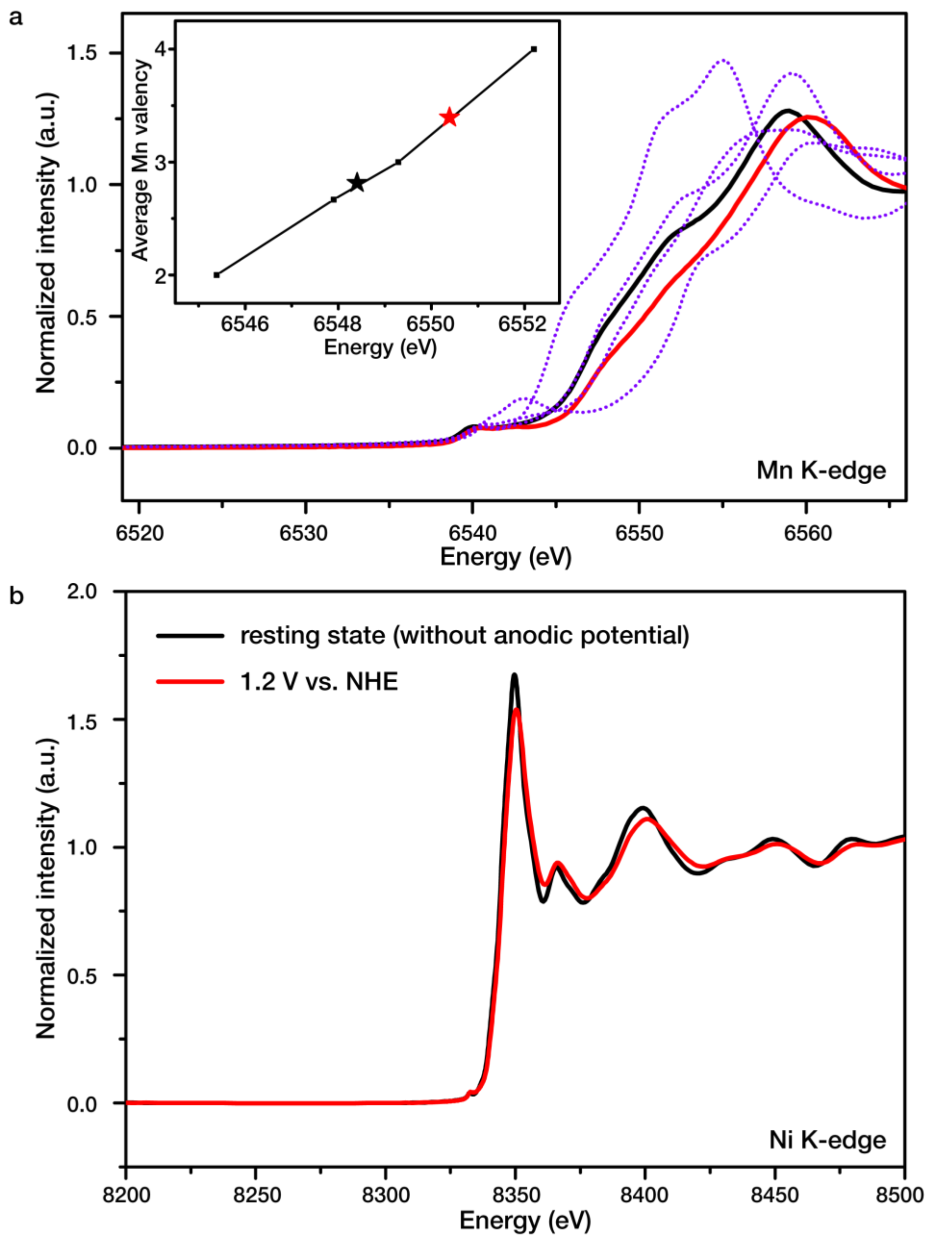

Supplementary Figure 3 | In-situ X-ray absorption near edge structure (XANES) spectroscopy analysis of the Ni-Mn_3O_4 NPs/NiO sample. a, Mn K-edge XANES spectra of the Ni-Mn_3O_4 NPs/NiO sample in 500 mM phosphate electrolyte (pH 7) with the spectra of reference manganese oxides powders. Inset figure shows the average oxidation state of Mn (black star: resting state without applied potential and red star: 1.2 V vs. NHE). **b**, Ni K-edge XANES spectra of the samples.

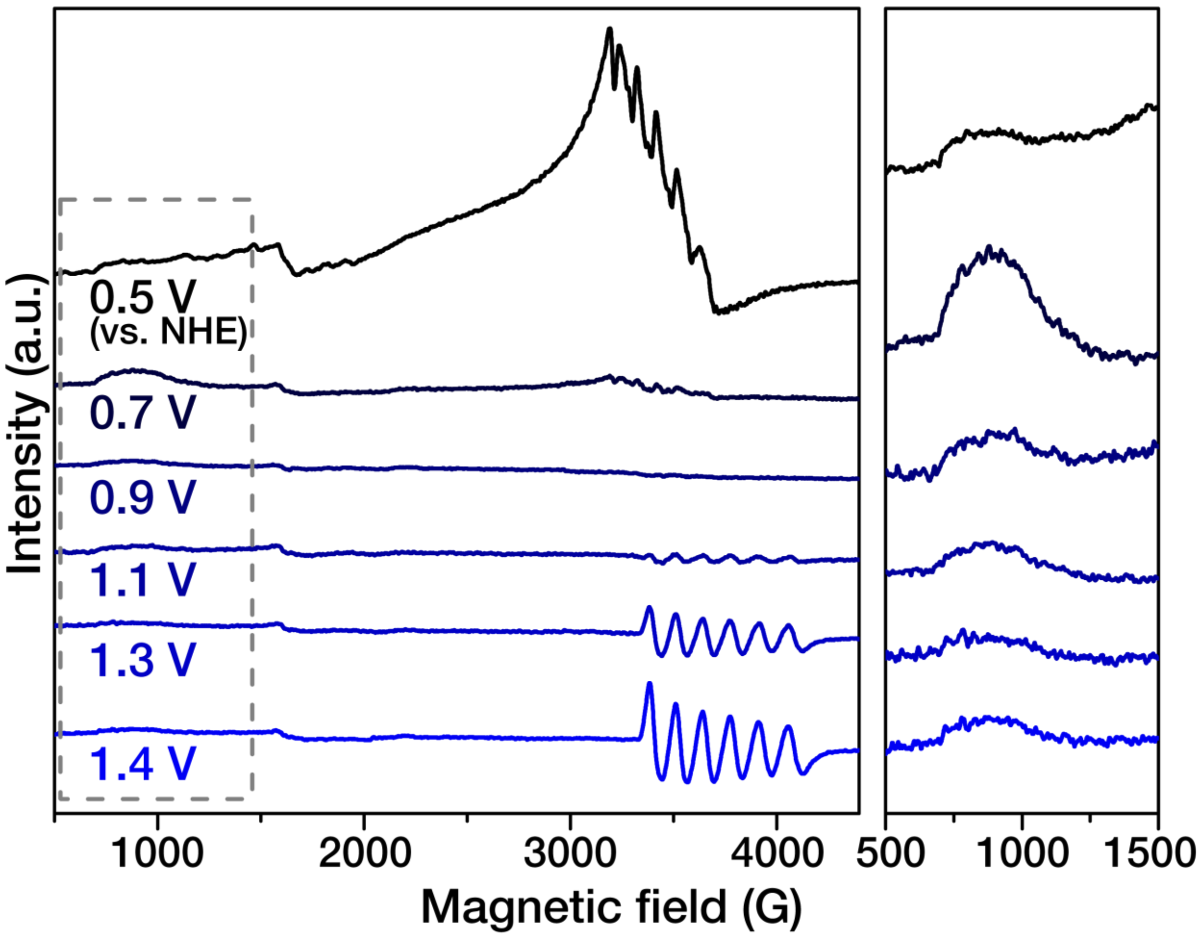

Supplementary Figure 4 | Potential dependent EPR spectra with wide scan in perpendicular mode. The right figure is the magnified graph of the grey rectangular region from the left graph. The broad feature near $g \sim 8$ in the low-field region is from the Mn(III) seen at $g \sim 8$ in parallel mode.

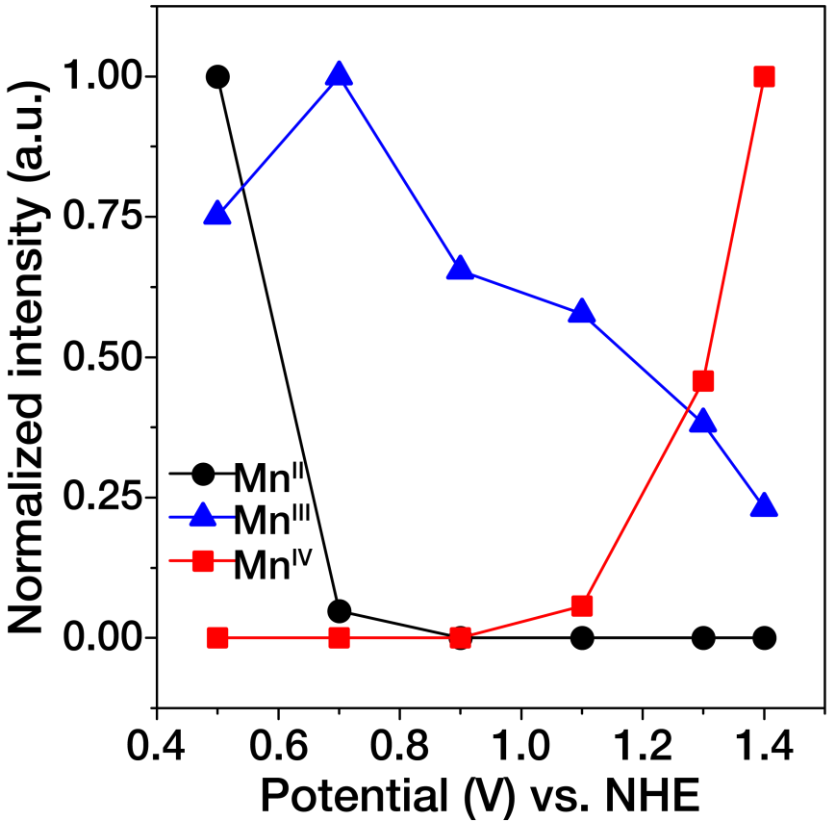

Supplementary Figure 5 | Normalized intensities of each Mn species depending on the applied potential.

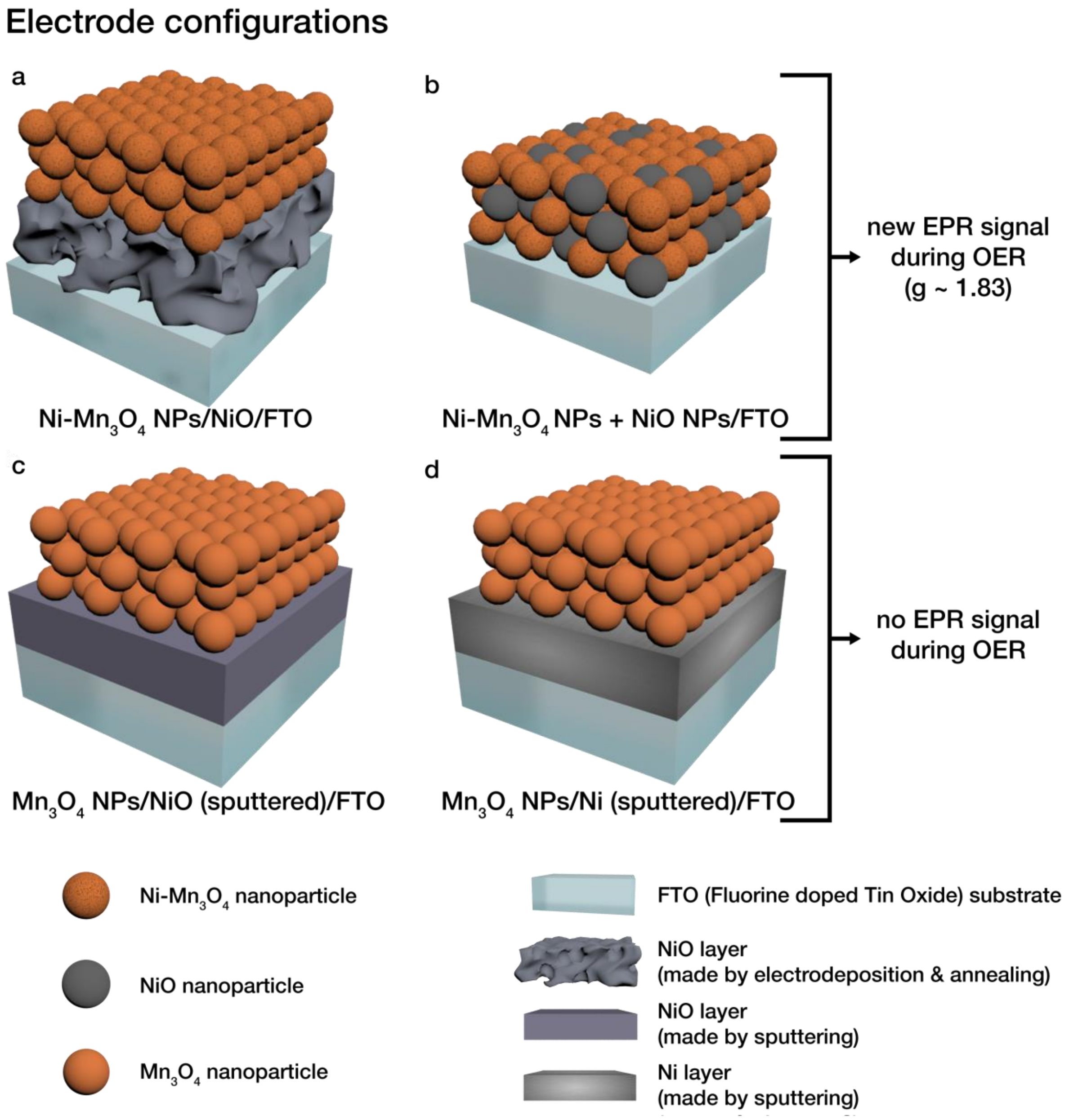

Supplementary Figure 6 | Various types of electrode configurations to find out the proper electrode configuration for effective Ni doping. Electrodeposited nickel hydroxide bottom layer (**a**) and mixed NiO NPs with Mn_3O_4 NPs (**b**) configurations show EPR signal at g ~1.83 during the electrolysis. No EPR signal is observed from the samples with sputtered NiO bottom layer (**c**) and sputtered Ni bottom layer (**d**) configurations during the electrolysis. All electrodes were prepared under the same thermal annealing condition (300 °C 5 hours, air) after the Mn_3O_4 NPs coating.

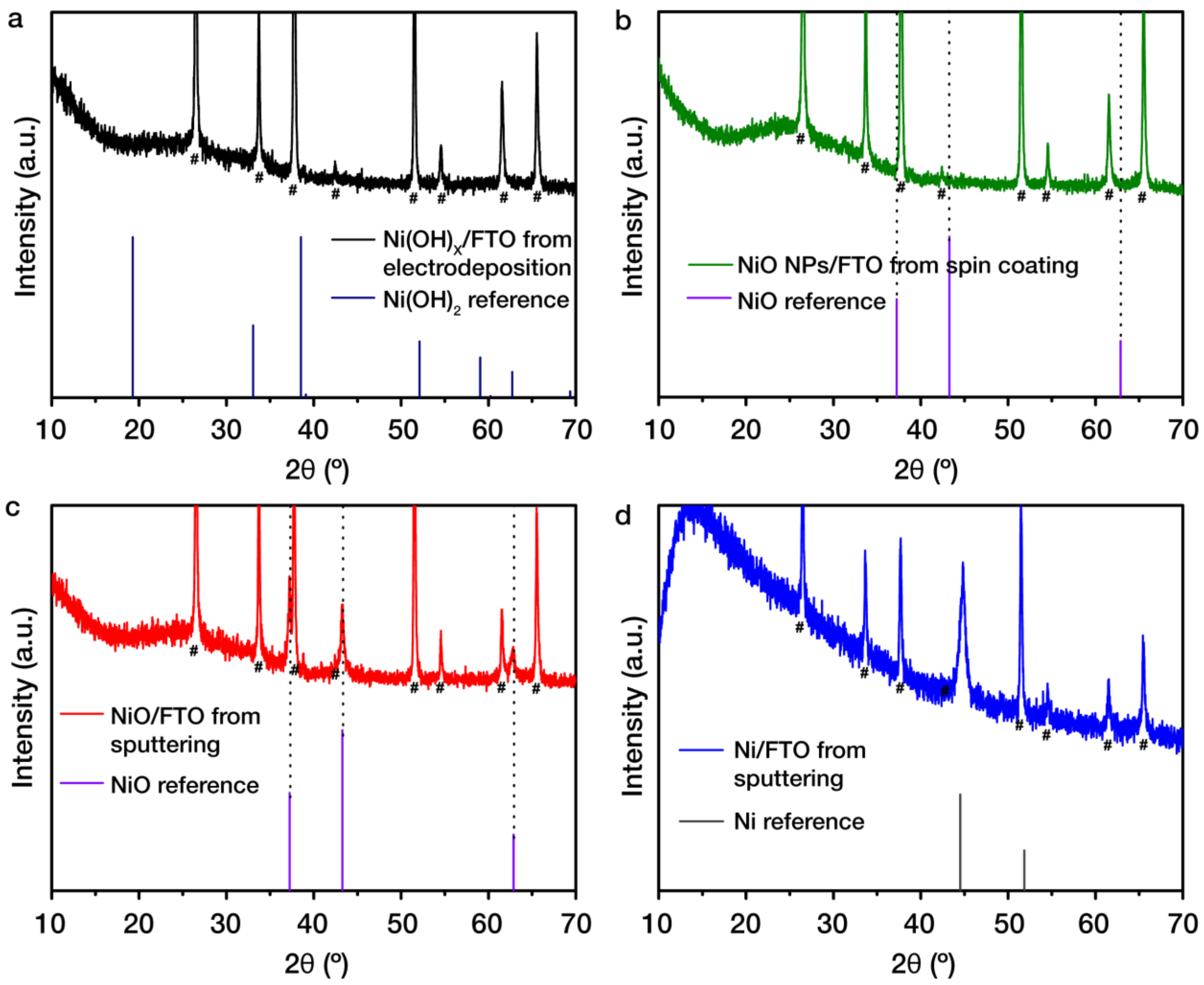

Supplementary Figure 7 | X-ray diffraction (XRD) patterns of prepared various electrodes. XRD spectra were measured from electrodeposited nickel hydroxide (**a**), NiO nanoparticles (NPs) (**b**), sputtered NiO (**c**), and sputtered Ni (**d**) respectively. # marks in each graphs indicate the diffraction peak from the FTO substrate. No distinct XRD peak is observed from nickel hydroxide (**a**) and NiO NPs (**b**) samples except for that of FTO substrate, indicating low crystallinity.

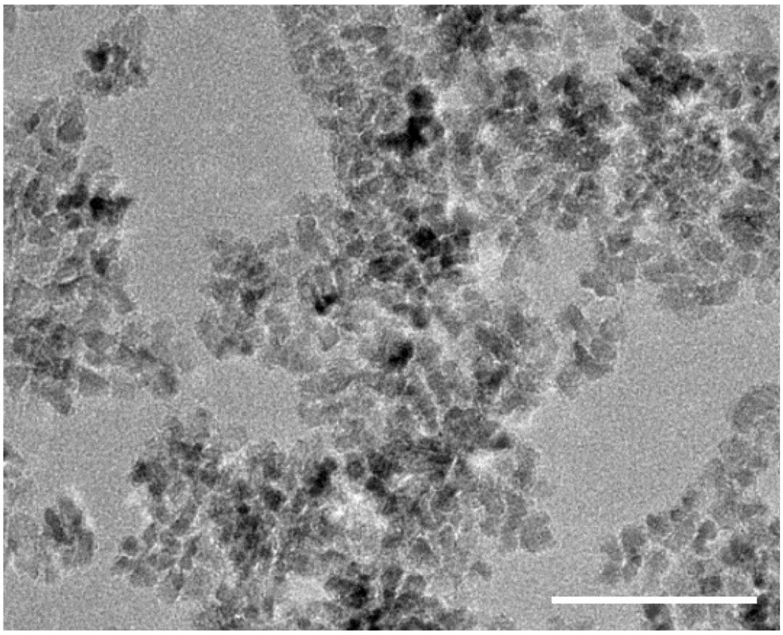

Supplementary Figure 8 | TEM image of synthesized NiO nanoparticles. Scale bar is 50 nm.

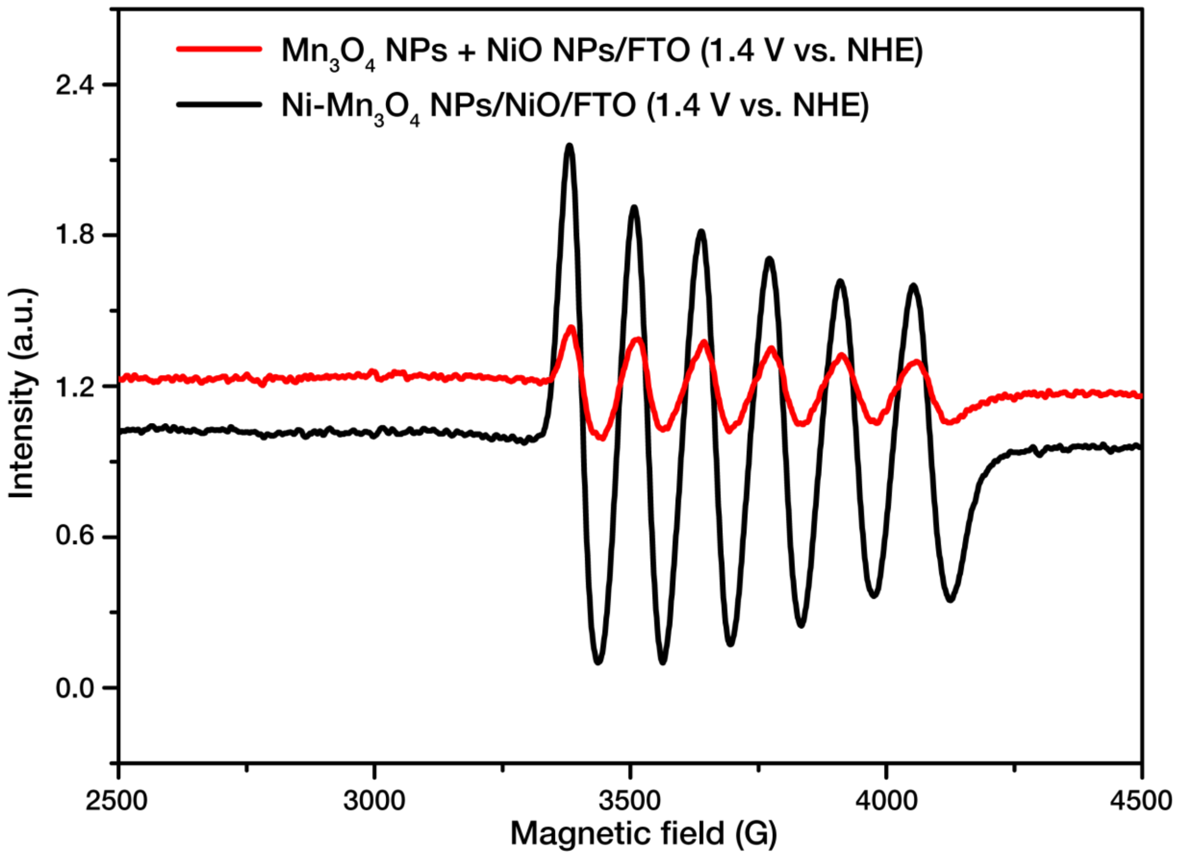

Supplementary Figure 9 | Continuous wave X band EPR spectra of prepared electrodes during electrolysis. Same EPR signal (g_{eff} ~1.83) was observed from different types of electrode. Electrolysis was performed in 500 mM phosphate electrolyte (pH 7) at 1.4 V (vs. NHE).

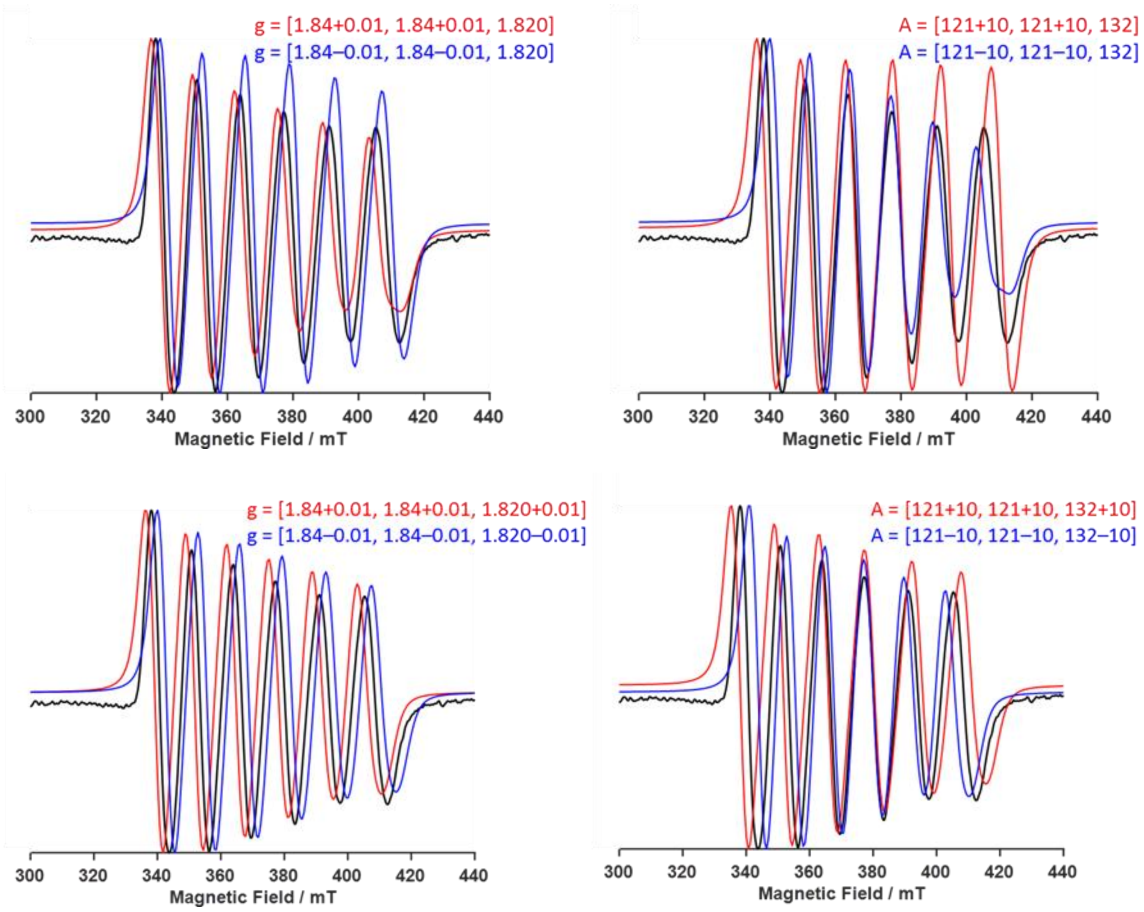

Supplementary Figure 10 | Simulated EPR spectra with the extreme simulation parameter values. Simulated spectrum is fairly sensitive by the change of the simulation parameters: $g = [1.84 \pm 0.01, 1.84 \pm 0.01, 1.82 \pm 0.01]$; $|A| = [121 \pm 10, 121 \pm 10, 132 \pm 10]$ G. The simulations are displayed with the extreme values (red in the highest value and blue in lowest value) within the error range, confirming the accuracy.

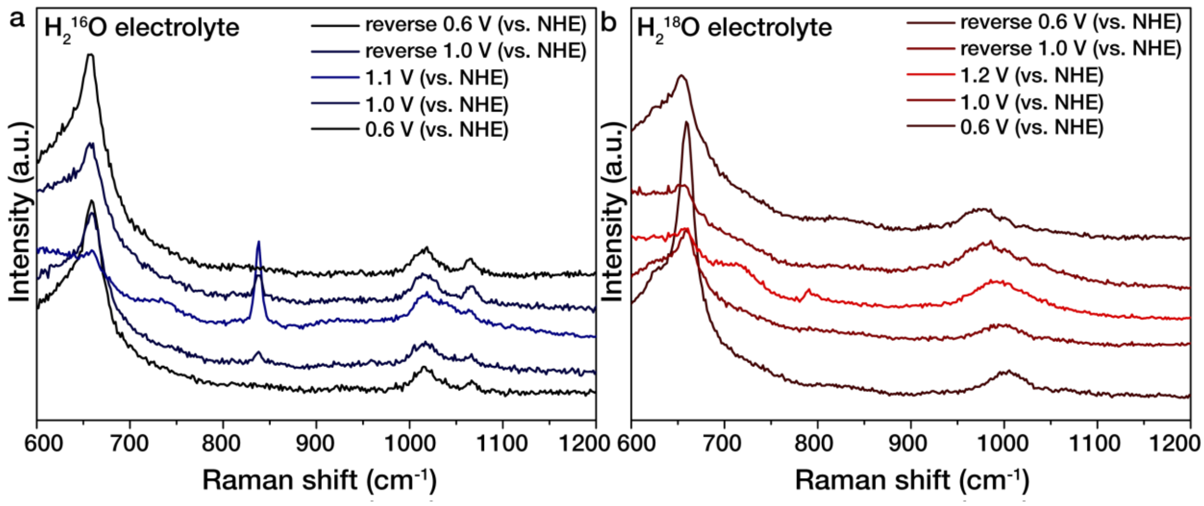

Supplementary Figure 11 | In-situ Raman spectra of Ni-Mn_3O_4 NPs/NiO/FTO. Raman spectra are observed under the 1 M $KHCO_3$ in $H_2{}^{16}O$ (**a**) and 1 M $KHCO_3$ in $H_2{}^{18}O$ (**b**). Applied potential is increased from 0.6 V (vs. NHE) to 1.1 V (vs. NHE) and then reversely decreased to 0.6 V (vs. NHE). Symmetric stretching vibrational mode of C–O from CO_3^{2-} (1066 cm^{-1}) and C–OH from HCO_3^- (1016 cm^{-1}) are clearly observed in the $H_2{}^{16}O$ electrolyte.[3] In the $H_2{}^{18}O$ electrolyte, only one broad peak from carbonate ion is observed, which is gradually red-shifted by the substitution of ^{18}O occurring as the measurement time passes. Note that, Raman peak at 837 cm^{-1} in $H_2{}^{16}O$ electrolyte can be assigned to soluble MnO_4^-.[4] The magenta-colored soluble MnO_4^- species are generally observed for manganese-based catalysts when the anodic potential is applied, which could ultimately lead to deactivation of the manganese-based electrodes.[5]

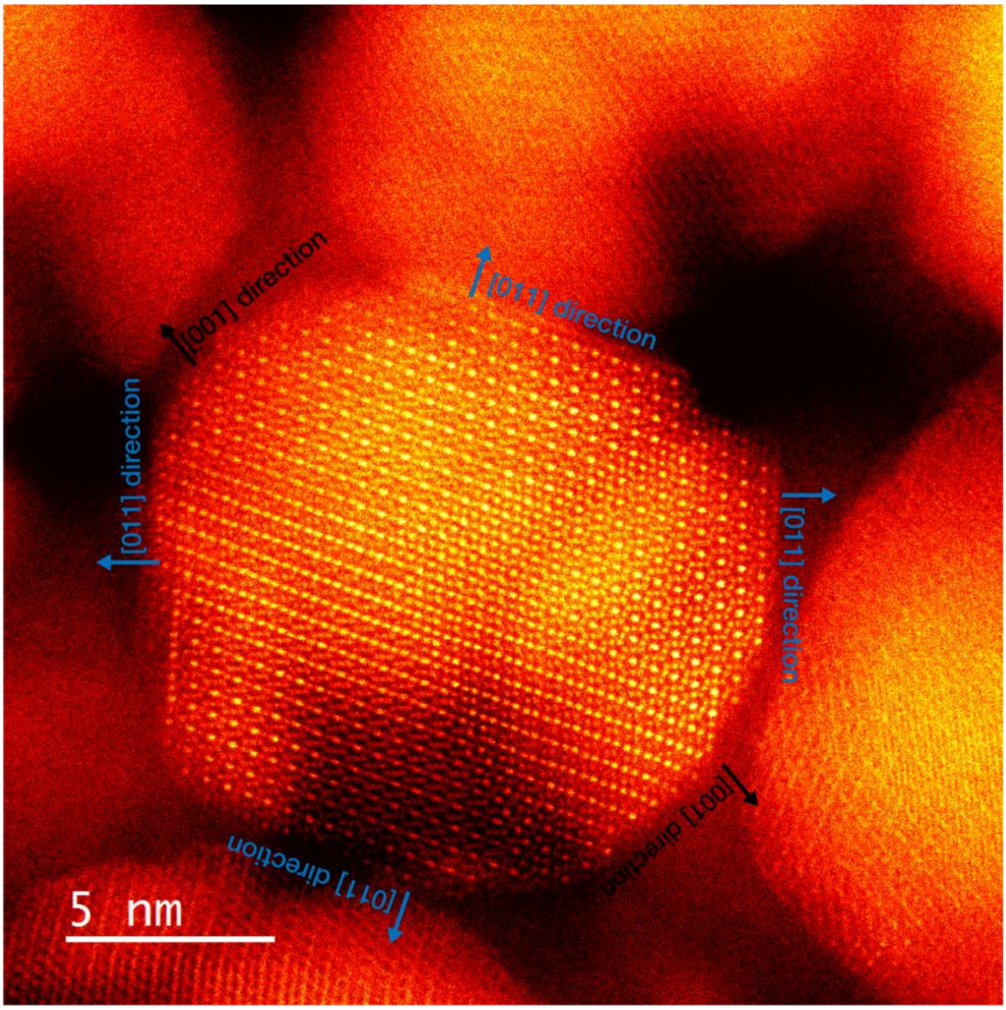

Supplementary Figure 12 | Atomic surface structure of Mn_3O_4 NPs from high-resolution high-angle annular dark field scanning transmission electron microscopy (HAADF STEM) image. The image viewed along the [100] zone axis. Surface of Mn_3O_4 NPs is constructed by the (001) facet and (011) facet with steps between two facets. Exposed high index facets are assigned as (x15), (x24), and (x31) (x =0, 1, 2…) assuming that the observed step structure in the image is maintained up to the depth direction.

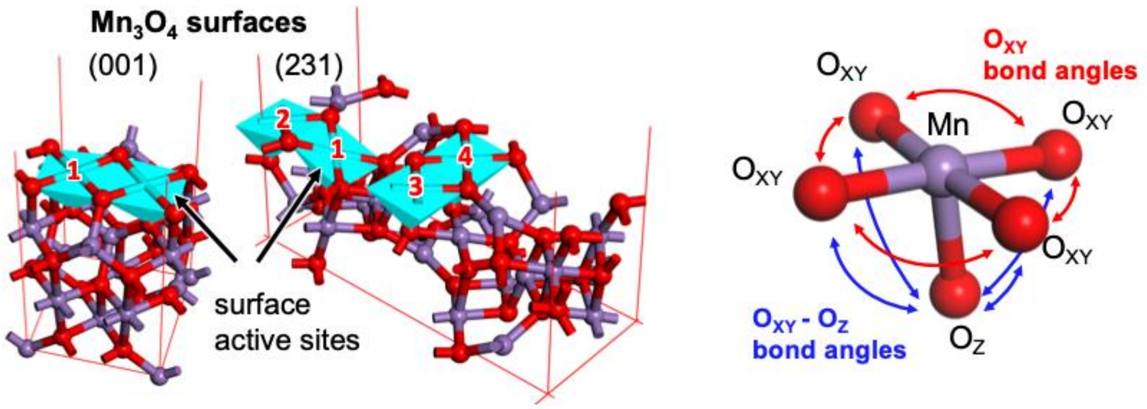

Supplementary Figure 13 | The structure of the surface active sites and the nomenclature of each atom.

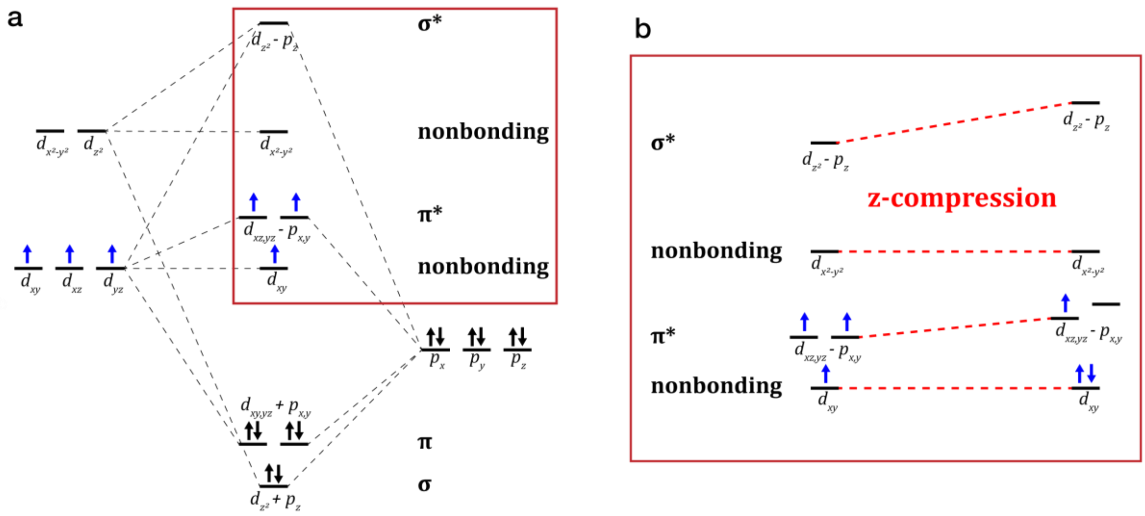

Supplementary Figure 14 | Schematic illustration of molecular orbital and electronic configuration. a, Electronic configuration of six-coordinate Mn(IV)-oxo (d^3) with pseudo-octahedral geometry. **b**, Ligand field splitting under the z-compression. For the pseudo-octahedral manganese, the frontier orbital of the Mn(IV)=O bond consists of the ligand field splitting of d-orbitals; three low-lying t_{2g}-type d_{xy} orbitals with non-bonding character and a $d_{xz/yz}$ orbital with π* antibonding character (axial field), and two e_g-type d_{z^2} orbitals with σ* antibonding character (axial) and a $d_{x^2-y^2}$ orbital with σ* antibonding character (equatorial field). Strengthened axial crystal field increases the energy gap between d_{xy} and $d_{xz/yz}$ and favors a low-spin configuration

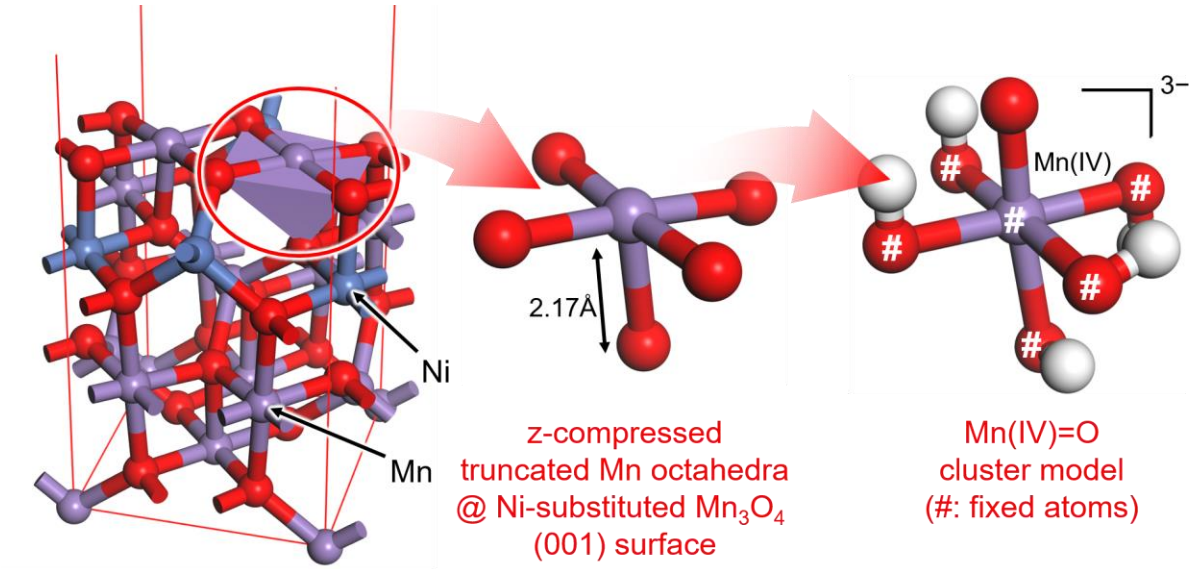

Supplementary Figure 15 | Procedure for sampling Mn octahedral single cluster model. The truncated Mn octahedral compressed along the z-axis by Ni-substitution at the Mn_3O_4 (001) surface. Surrounding oxygen atoms are hydroxylated to modulate the ligand strength. Only the hydrogen and oxygen adsorbate atoms are relaxed during structure optimization to keep its original ligand field.

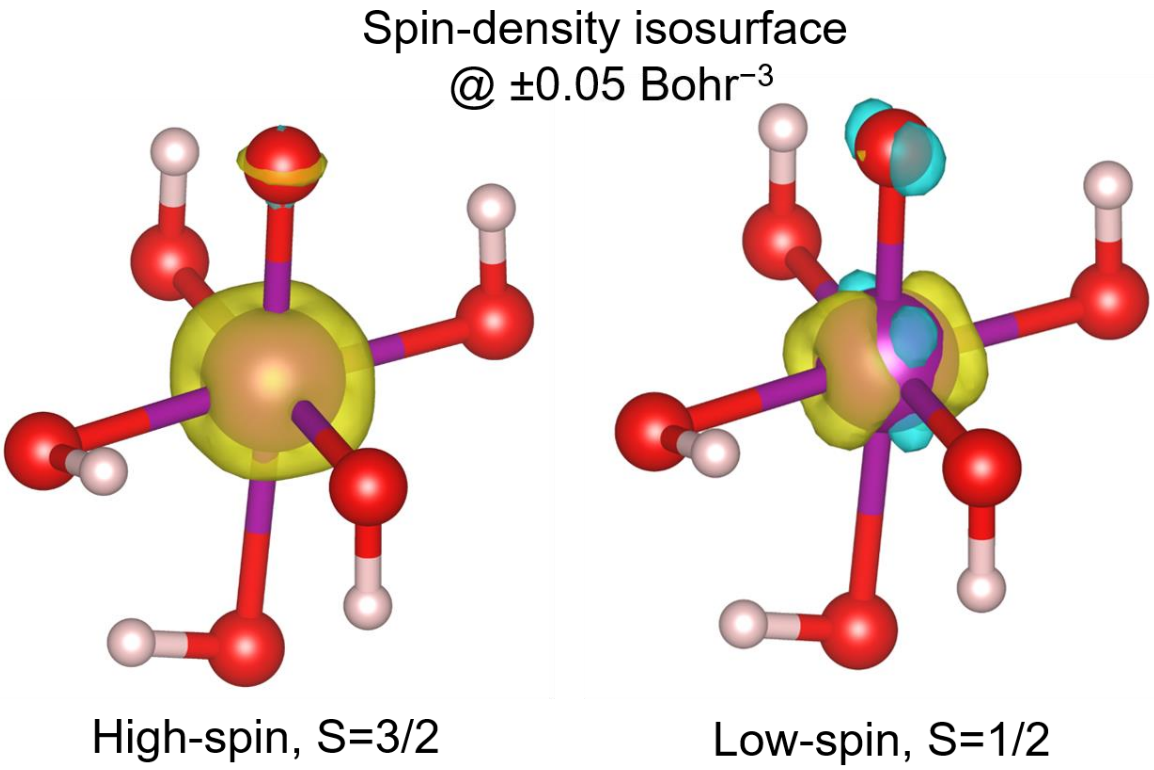

Supplementary Figure 16 | Spin density isosurface plot for high- and low-spin electronic structures of Mn(IV)=O cluster model.

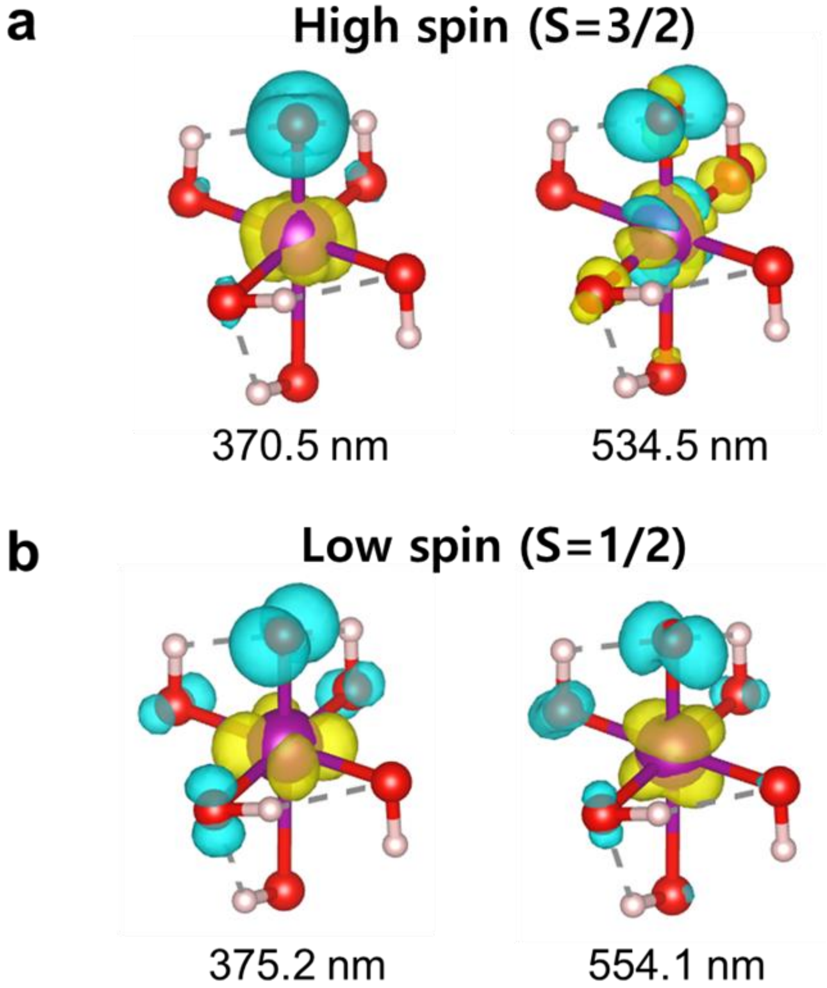

Supplementary Figure 17 | Electron density difference map for the transitions with the large oscillatory strength near 400 nm and 600 nm. a, High-spin Mn(IV)-oxo shows π-type ligand-to-metal charge transfer (LMCT) band at 370.5 nm and *d*-*d* transition band at 534.5 nm. **b,** Low-spin Mn(IV)-oxo shows π-type LMCT bands at 375.2 nm and 554.1 nm. Due to the broken degeneracy of d_{xz} and d_{yz} orbital, which form π and π* orbitals by hybridizing with p_x and p_y orbitals, low-spin Mn(IV)-oxo can have different energies for the π-type LMCT.

	C_{dl} (mF)	ECSA (cm^2)	i at 1.3 V (mA)	Number of active sites (Mn sites cm^{-2})	Turnover frequency at 1.3 V (s^{-1})
Ni-Mn_3O_4 NPs/NiO/FTO	3.38	84.6	1.995	1.134E15	0.0325
Mn_3O_4 NPs/FTO	1.39	34.8	0.705	1.134E15	0.0279
NiO/FTO	1.12	28.0	0.105	1.444E15	0.00405

Supplementary Table 1 | Calculated turnover frequency for prepared electrodes. The electrochemically active surface area is calculated from measured double-layer capacitance. The number of active sites is calculated using the crystallographic data of Mn_3O_4 for Ni-Mn_3O_4 NPs/NiO/FTO and Mn_3O_4 NPs/FTO samples, and NiO for NiO/FTO sample. The unit cell of Mn_3O_4 contains 12 Mn atoms and the volume 314.3 $Å^3$. The calculated number of surface Mn atoms is 1.134×10^{15} Mn atoms cm^{-2}.[6] The unit cell of NiO contains 4 Ni atoms and the volume 72.87 $Å^3$. The calculated number of surface Ni atoms is 1.444×10^{15} Ni atoms cm^{-2}.[7] Turnover frequencies at 1.3 V (vs. NHE) are calculated by the following equation with the assumption that all metal atoms on the surface serve as the active sites.

$$\text{turnover frequency} = \frac{(\text{current density at 1.3 V vs. NHE})}{4 \times \text{Faraday constant} \times (\text{number of active sites})}$$

	Mn-O_{XY} distances (Å)				Mn-O_Z distance (Å)	O_{XY}-Mn-O_{XY} angles (°)				O_{XY}-Mn-O_Z angles (°)			
(001)	1.957	1.957	1.924	1.924	2.301	98.67	98.67	81.38	81.28	97.35	97.35	82.35	82.35
(001)-Ni	1.996	1.924	1.924	1.918	2.170	98.30	96.27	82.89	82.68	97.65	96.07	86.70	79.56
$(231)_1$	1.973	1.955	1.954	1.910	2.316	97.73	95.67	84.85	81.89	105.67	103.97	77.02	75.84
$(231)_1$-Ni	2.000	1.978	1.930	1.926	2.215	103.76	87.40	84.79	83.96	105.10	103.72	80.59	79.54
$(231)_2$	2.135	1.986	1.912	1.892	2.127	97.19	93.93	85.13	83.59	100.88	98.85	85.82	82.50
$(231)_2$-Ni	2.199	1.979	1.961	1.897	2.076	95.91	95.48	86.37	82.16	98.40	96.69	84.81	84.38
$(231)_3$	2.068	2.019	2.009	1.977	2.175	103.74	97.95	79.50	78.88	98.94	97.68	88.86	80.35
$(231)_3$-Ni	2.080	2.029	1.989	1.974	2.141	104.67	97.77	79.22	78.09	97.42	96.87	89.16	85.18
$(231)_4$	2.077	2.068	2.013	1.971	2.138	102.97	100.00	79.80	77.37	96.74	93.89	91.27	81.86
$(231)_4$-Ni	2.071	2.053	2.015	1.966	2.084	103.74	100.18	79.52	76.45	98.83	93.74	93.73	81.93

Supplementary Table 2 | Calculated bond distances and angles of truncated surface Mn active sites before and after the Ni substitution. Ni substituted structures were chosen from the maximally compressed axial Mn–O bond cases in Figure 6c.

Supplementary References

1 Kanan, M. W. & Nocera, D. G. In situ Formation of an Oxygen-Evolving Catalyst in Neutral Water Containing Phosphate and Co^{2+}. *Science* **321**, 1072-1075 (2008).

2 Huynh, M., Shi, C., Billinge, S. J. & Nocera, D. G. Nature of activated manganese oxide for oxygen evolution. *J. Am. Chem. Soc.* **137**, 14887-14904 (2015).

3 Rudolph, W. W., Irmer G. & Königsberger, E. Speciation studies in aqueous HCO_3^-–CO_3^{2-} solutions. A combined Raman spectroscopic and thermodynamic study. *Dalton Trans.*, 900-908 (2008).

4 Kiefer, W. & Bernstein, H. J. Resonance Raman Effect in MnO_4^- with 5145 Å laser excitation. *Chem. Phys. Lett.* **8**, 381-383 (1971).

5 Li, A. et al. Stable Potential Windows for Long-Term Electrocatalysis by Manganese Oxides Under Acidic Conditions. *Angew. Chem. Int. Ed.* **58**, 5054-5058 (2019).

6 Jarosch, D. Crystal Structure Refinement and Reflectance Measurements of Hausmannite, Mn_3O_4. *Mineralogy and Petrology* **37**, 15-23 (1987).

7 Wyckoff, R. W. G. *Crystal Structures Vol. 1*. (Interscience Publishers, New York, 1963).
