# Supplementary material for: Spectroscopic capture of a low-spin Mn(IV)-oxo species in Ni–Mn3O4 nanoparticles during water oxidation catalysis
Source: Nat Commun. 2020 Oct 16;11:5230. doi: 10.1038/s41467-020-19133-w (PMC7567882; doi:10.1038/s41467-020-19133-w)
Supplement: Supplementary file 2 — Peer Review File [file 41467_2020_19133_MOESM2_ESM.pdf]

Reviewer #1 (Remarks to the Author):

There is a lot to like about this paper. I did want to point out a few things.

The spectra vs potential of Fig 3a are really interesting. They do what they can with the CW spectra and calculations to make the case for the low spin Mn(IV) assignment, and they have their susceptibility data. But what is missing and seems compelling to do in my opinion is some pulse EPR study using one of the Rabi oscillation sequences to show the S=1/2 nature of the low g-value signal. This should be easy, and they have the S=5/2 "standard" right there in the same series of samples. This would be a clean and elegant and direct way to confirm their central point.

I am less keen on the parallel mode data of Fig 3b. The trend they discuss in the text for the rise of the putative Mn(III) signal are barely noticeable, with almost as much "Mn(III)" before potential as in the higher potential samples. They don't show the perp mode over the same field values, what is happening there? I am not at all convinced this is Mn(III) or at least all Mn(III) as S=5/2 Mn(II) can show low field parallel mode signals. This section needs more work. Or it should be removed.

When talking about other high valent transition metal oxo water oxidation catalysts, and the Fe/Ni systems in particular, I would think they would want to touch on the Gray/Hunter/colleagues evidence for ferrate species, which is very relevant to that part of the introduction discussion.

Reviewer #2 (Remarks to the Author):

The manuscript by Park and co-workers describes the spectroscopic characterization of a low-spin (S = ½) Mn(IV)-oxo species. This species is observed for Ni-doped Mn3O4 nanoparticles on a layer of NiO. The Ni-doped Mn3O4 nanoparticles also give rise to a lower overpotential for the oxygen-evolution reaction as compared to Mn3O4 alone. As far as I am aware, a low-spin state is unprecedented for a Mn(IV)-oxo complex, although a low-spin Mn(IV)-nitride has been reported. In addition, the EPR signal for this low-spin Mn(IV) complex is very different than any that has been reported previously (to the best of my knowledge). Thus, this new intermediate seems exciting and is quite novel. Given the importance of Mn(IV)-oxo species in biological and synthetic catalysts (most pertinently the oxygen-evolving complex of photosystem II), this finding should be of interest to a wide audience.

However, there are also several factors that moderate my enthusiasm for this work. First, and most importantly, there is no direct evidence that the low-spin Mn(IV) species is a Mn(IV)-oxo. There needs to be some vibrational data or other piece of evidence to demonstrate the presence of a terminal oxo ligand. In small-molecule chemistry, there are many examples of Mn(IV) complexes lacking oxo ligands. In these cases the EPR spectra are still distinct from that seen here, so the EPR signal is still novel, but the importance of this signal is less novel if it does not arise from a Mn(IV)-oxo species.

Second, the catalytic relevance of the low-spin Mn(IV) species is unclear. Is the low-spin Mn(IV) center truly an on-path intermediate or just a species that accumulates at very high potentials? Why should the low-spin Mn(IV) species be better suited for oxygen-evolution than the high-spin analogue?

Overall, I believe this work is potentially suitable for Nature Communications. However, the authors should address the major items raised above, as well as other points discussed below.

Major Items
In the abstract it is stated that the low-spin Mn(IV) signal evolves during the “stepwise transition from the Jahn-Teller-distorted Mn(III).” In addition, on line 163, the authors state that the Mn(III) signal slightly decreases as the anodic potential increased. While the low-spin Mn(IV) signal clearly increases in intensity as the potential is increased, there does not appear to be a concomitant decrease in the

Mn(III) EPR signal. To demonstrate that one species converts to the other, as stated in the abstract, the Mn(IV) and Mn(III) signals should be quantified (by integration) and this quantity should be plot versus the applied potential. This plot could provide evidence if the Mn(III) is being consumed as the low-spin Mn(IV) is being formed.

The computational work is not completely described. First, the authors have used computations to model the Mn(IV)-oxo species as an octahedral complex, but the most relevant point of comparison (the low-spin Mn(IV)-nitride) is a tetrahedral complex. Why was a tetrahedral geometry not considered? Second, while A values were calculated, g values were not. Given the highly unusual g values observed experimentally, it is odd that the g values were not calculated and compared with experiment. Third, the metric parameters for the low-spin Mn(IV) complex are of interest. The authors should discuss bond lengths, angles, etc., especially in relation to the high-spin counterpart. Do the computations offer insight into the structural basis for the stabilization of the low-spin state?

Minor Items
Page 4: The oxygen-evolving complex is more commonly referred to as the Mn4Ca cluster (as is done in the abstract), rather than the MnCa cluster, to denote the Mn nuclearity.

Page 7, lines 139 – 141: The assignment of the 400 nm and 650 nm electronic absorption features for the low-spin Mn(IV) species to LMCT and d-d transitions, respectively, seems at odds with the relative intensities of these features. In fact, the intensities are inverted compared to that expected and compared to that observed for the intensities of the LMCT and d-d transitions for the putative high-spin Mn(IV)-oxo species shown in Figure 2e. What explanation is there for the big change in the relative intensities of these features?

Page 9, lines 208 – 209: For the more general audience of Nature Communications, the Vvib and VL terms should be more completely described. In addition, the EJPT term (line 224) should be defined more explicitly. I presume this is the splitting between the xz and yz orbitals, but this is not stated.

Page 12, lines 266 – 273: The spectral assignments from the TD-DFT computations, as LMCT or d-d bands, are incomplete. The computations provide precise information on the orbitals involved in these excitations, and this information should be provided. For example, the S = ½ Mn(IV) ion will have many d-d transitions. Which are observed in the visible region for the TD-DFT computations? Are the LMCT transitions of pi- or sigma-type?

The [e3] and [b2e1] nomenclature (using subscripts) is used throughout the paper to represent electron configurations. However, the standard nomenclature is to use superscripts to note orbital occupancy; i.e., (e)3 and (b)2(e)1 (using superscripts). This approach has the advantage that a (b)2 configuration can be distinguished from a b2 Mulliken symbol.

Reviewer #3 (Remarks to the Author):

In this very interesting manuscript, the authors report for the first time on new intermediates during the water oxidation reaction of manganese-oxide based electrocatalysts, assigned as a low-spin Mn(IV)-oxo species. To capture the reactive intermediate, they have used several characterization techniques, such as: in-situ potential dependent UV-Vis spectroscopy, ex-situ EPR under electrocatalytic operation conditions, and DFT calculations. These important findings should pave the way to widen our understanding regarding the mechanisms of electrochemical water oxidation. In addition, the gained insights will aid in the future design of highly efficient catalysts for a wide range of applications. As such, I recommend publication in Nature Communications, subject to addressing several points.

1) The authors should discuss the reasons behind the motivation to run water oxidation reaction at neutral pH, rather than in alkaline solutions where known catalysts exhibit much higher catalytic activity.

2) Is there any other possible characterization tools that could be utilized in the future for catalytic intermediate capture?

3) Can the authors compare the water oxidation intrinsic activity (TOF) for their characterized samples (Mn_3O_4, Ni-Mn_3O_4, and NiO)?

Response to reviewers

We appreciate your careful review of our manuscript and the reviewer's valuable comments. Reviewer #1 highly valued our paper, stating, *"There is a lot to like about this paper."* Reviewer #3 also expressed support for the publication of this manuscript with the following comment: *"These important findings should pave the way to widen our understanding regarding the mechanisms of electrochemical water oxidation. In addition, the gained insights will aid in the future design of highly efficient catalysts for a wide range of applications"*. Importantly, reviewer #2 suggested the addition of vibrational data to demonstrate the presence of terminal oxo species and a more detailed description of the computational work.

During the revision, we newly performed an in-situ Raman spectroscopy experiment to provide direct evidence for the generation of terminal oxo species. The detailed changes in the revised manuscript are as follows. (1) New figures discussing the in-situ Raman experimental results were added in the main text to prove the generation of terminal oxo species (Figure 6 and Supplementary Figure 11). (2) We also added all detailed metric parameters for the low-spin Mn(IV)-oxo complex from the computational calculation in Supplementary Information (Supplementary Figure 13). (3) We revised the discussion on the spectroscopy data with more detailed descriptions. The description of the UV-Vis spectrum of Ni-Mn_3O_4 NPs was revised and the calculation result for the electronic transition was added (Supplementary Figure 17). Additionally, the new figure was added to clearly show the changes in the EPR intensities according to the applied potential (Figure 3 and Supplementary Figure 5). (4) Turnover frequencies of each electrocatalyst (Ni-Mn_3O_4 NPs/NiO, Mn_3O_4 NPs, and NiO) were added to compare the intrinsic activities for water oxidation reaction (Supplementary Table 1). The turnover frequency was derived by measuring the electrochemically active surface area (Supplementary Figure 1). (5) More discussion about the motivation for water oxidation reaction at neutral pH condition and the active oxo species on Fe/Ni electrocatalysts was added to the introduction.

We are grateful to reviewers for their valuable comments that helped us to improve the clarity of our manuscript. Now, we are optimistic that the reviewers will now agree that the previous weaknesses were completely revised. Our point-by-point responses to reviewers' comments are as followed. New texts added in the revised manuscript are indicated in yellow, and new texts in the supplementary information are indicated in blue.

Reviewer 1

There is a lot to like about this paper. I did want to point out a few things.

[Response]

We appreciate the reviewer's valuation and positive comment on our manuscript. The revised manuscript could be improved by following your suggestions. Additionally, we added in-situ Raman results during the revision to provide direct evidence for the generation of terminal Mn(IV)-oxo species. Below is the detailed point-by-point response for your suggestion.

[Reviewer 1-Comment 1] *The spectra vs potential of Fig 3a are really interesting. They do what they can with the CW spectra and calculations to make the case for the low spin Mn(IV) assignment, and they have their susceptibility data. But what is missing and seems compelling to do in my opinion is some pulse EPR study using one of the Rabi oscillation sequences to show the S=1/2 nature of the low g-value signal. This should be easy, and they have the S=5/2 "standard" right there in the same series of samples. This would be a clean and elegant and direct way to confirm their central point.*

[Response]

We thank the reviewer for the insightful comments. As the reviewer suggested, the ground spin state of an EPR signal can be determined by electron spin nutation using one of the Rabi oscillation sequences since the optimal length of microwave pulses is defined by the effective spin state of the species being measured. Thus, electron spin nutation experiments would provide compelling evidence for the new EPR signal assignment as the S = 1/2, low-spin Mn(IV). In fact, we tried to do spin nutation experiments; however, we could not get any spin echo from this sample, which is pre-requisite to do electron spin nutation experiments. We thought that the fast relaxation behavior of the nanoparticles might be a culprit; thus, we could not do any pulse experiments on this sample. Nevertheless, as the reviewer pointed out, we carefully calculated and described this unusual Mn(IV) EPR signal along with magnetization data to make an assignment S = 1/2, Mn(IV).

[Reviewer 1-Comment 2] *I am less keen on the parallel mode data of Fig 3b. The trend they discuss in the text for the rise of the putative Mn(III) signal are barely noticeable, with almost as much*

"Mn(III)" before potential as in the higher potential samples. They don't show the perp mode over the same field values, what is happening there? I am not at all convinced this is Mn(III) or at least all Mn(III) as S=5/2 Mn(II) can show low field parallel mode signals. This section needs more work. Or it should be removed.

[Response]

We appreciate the reviewer's valuable comments. In fact, we made a mistake in plotting the EPR data in the main figure; the intensities of each spectrum were modified for visibility. As shown in the revised manuscript and Figure 3, we plotted the EPR data without the intensity modification, which shows that the Mn(III) signal's intensity is changed depending on the applied potential. Besides, we added the plot of the normalized intensity changes of the Mn EPR signal for each oxidation state versus the applied potential to show the changes of the intensity of EPR signals' intensity as a function of the potential more clearly (Supplementary Figure 5). Regarding the EPR spectra of low field region in perpendicular mode, we showed the perpendicular mode EPR spectra over the wide range of the magnetic fields (from 500 G up to ~4500 G) in the supplementary information (Supplementary Figure 4). At the low field region in perpendicular mode (*g*~8.0), a small Mn(III) signal is also seen. When the Mn(III) signal is intense, we usually observe it in the same field region, even in perpendicular mode (Supplementary Figure 4). This is because of the not perfect match of the $B_0 \perp B_1$ field in the resonator. The Mn(III) ($S = 2$, d^4) signal, which can be seen with the parallel mode is previously well-characterized [*J. Am. Chem. Soc.* 139 (6), 2277 (2017), *Sci. Rep.* 5, 10279 (2015), *ACS Catal.* 5 (8), 4624 (2015)]. This is because of the appropriate magnitude of the ZFS of Mn(III). Thus it is clear that the observed signal at *g*~8 in parallel mode arises from Mn(III). Overall, we successfully monitored the sample's the oxidation state changes depending on the applied potentials from the EPR analysis.

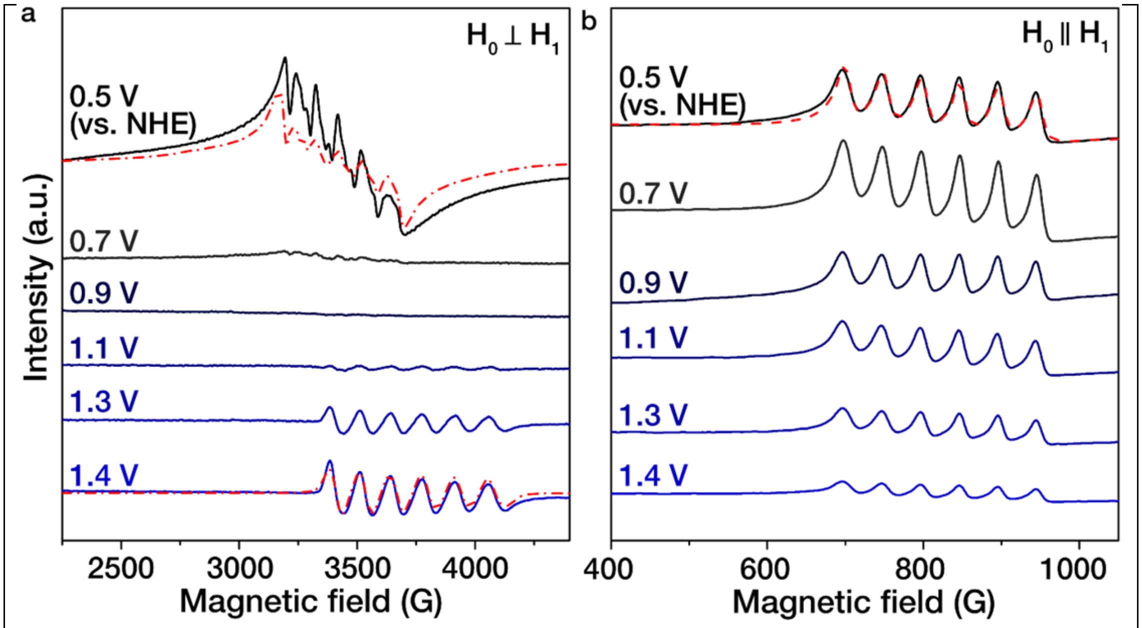

Figure 3 | Measured and simulated potential dependent EPR spectra. **a** and **b**, Potential-dependent X-band CW-EPR spectra of Ni-Mn_3O_4 NPs/NiO in perpendicular (**a**) and parallel (**b**) modes. The dashed red lines are spectral simulations that are well matched with the experimental curves.

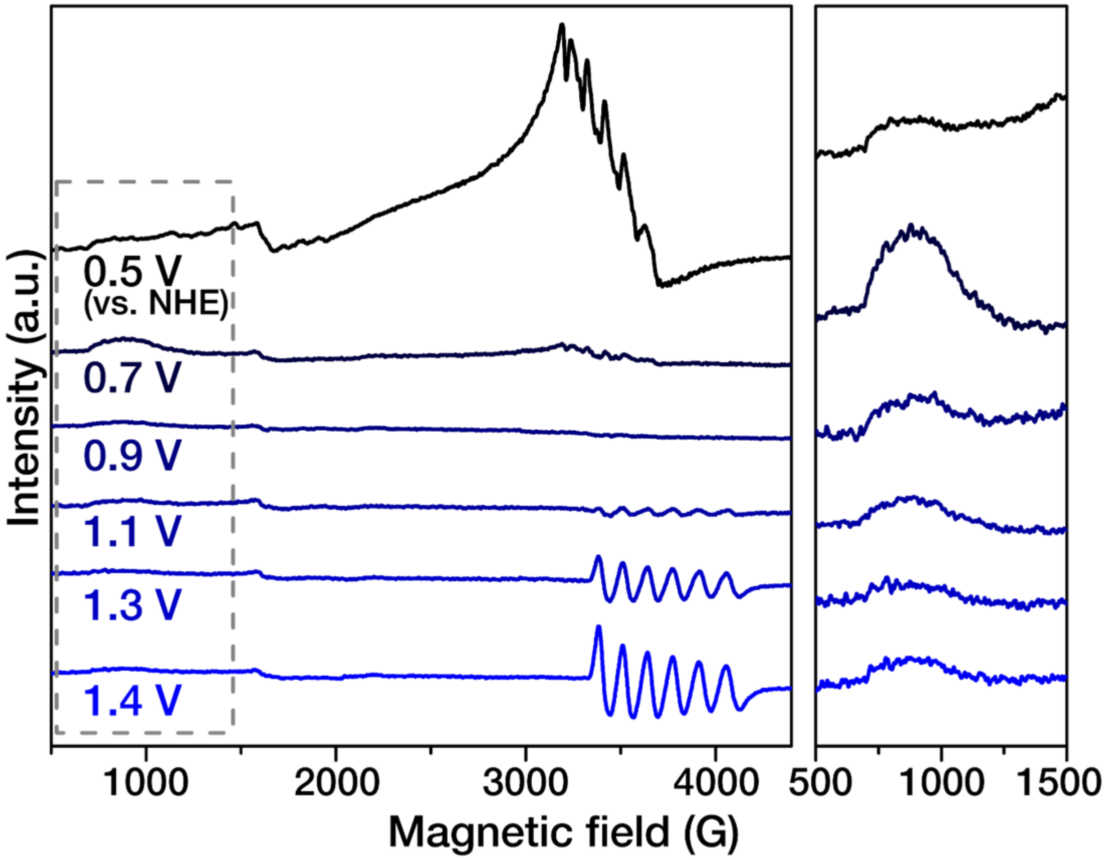

Supplementary Figure 4 | Potential dependent EPR spectra with wide a scan in perpendicular mode. The right figure is the magnified graph of the grey rectangular region from the left graph. The broad feature near *g* ~ 8 in the low-field region is from the Mn(III) seen at *g* ~ 8 in parallel mode.

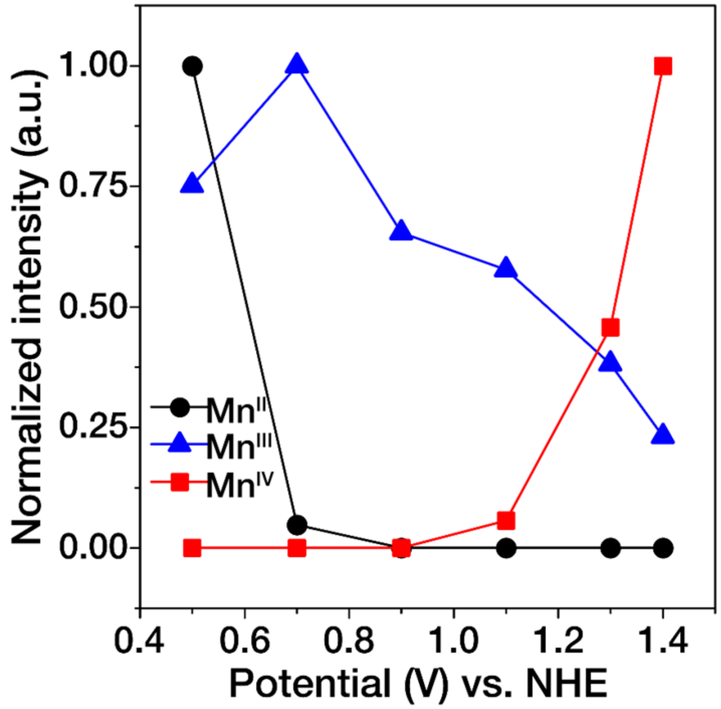

Supplementary Figure 5 | Normalized intensities of each Mn species depending on the applied potential.

[Reviewer 1-Comment 3] *When talking about other high valent transition metal oxo water oxidation catalysts, and the Fe/Ni systems in particular, I would think they would want to touch on the Gray/Hunter/colleagues evidence for ferrate species, which is very relevant to that part of the introduction discussion.*

[Response]

We thank the reviewer for pointing out an important citation that was missing in our previous manuscript. We added the paper as a reference and revised the introduction part. Shown below is the revised discussion that has been added to the manuscript

Interestingly, Gray and colleagues experimentally capture cis-dioxo-Fe(VI) intermediate species from NiFe hydroxide catalyst in a nonaqueous electrolyte.[15] Limitation of the substrate (H_2O and OH^-) in nonaqueous media permits the accumulation of active intermediate species, which allows

the use of various in-situ spectroscopic measurements (infrared, UV-Vis, Raman, luminescence, and Mössbauer spectroelectrochemistry). They suggested that the O–O bond formed through the internal redox rearrangement mechanism of cis-dioxo-Fe(VI) to Fe(IV)-peroxide intermediate species.

Reviewer 2

The manuscript by Park and co-workers describes the spectroscopic characterization of a low-spin (S=1/2) Mn(IV)-oxo species. This species is observed for Ni-doped Mn3O4 nanoparticles on a layer of NiO. The Ni-doped Mn3O4 nanoparticles also give rise to a lower overpotential for the oxygen-evolution reaction as compared to Mn3O4 alone. As far as I am aware, a low-spin state is unprecedented for a Mn(IV)-oxo complex, although a low-spin Mn(IV)-nitride has been reported. In addition, the EPR signal for this low-spin Mn(IV) complex is very different than any that has been reported previously (to the best of my knowledge). Thus, this new intermediate seems exciting and is quite novel. Given the importance of Mn(IV)-oxo species in biological and synthetic catalysts (most pertinently the oxygen-evolving complex of photosystem II), this finding should be of interest to a wide audience.

[Response]

We truly appreciate the reviewer for consideration on our work and insightful comments. In accordance with the comments, we have revised the manuscript in a point-by-point manner.

[Reviewer 2-Comment 1] *First, and most importantly, there is no direct evidence that the low-spin Mn(IV) species is a Mn(IV)-oxo. There needs to be some vibrational data or other piece of evidence to demonstrate the presence of a terminal oxo ligand. In small-molecule chemistry, there are many examples of Mn(IV) complexes lacking oxo ligands. In these cases the EPR spectra are still distinct from that seen here, so the EPR signal is still novel, but the importance of this signal is less novel if it does not arise from a Mn(IV)-oxo species.*

[Response]

Thank you for your suggestion. We agree that vibrational data was missing in our previous manuscript, which is the direct evidence for oxo species. Considering the importance of the vibrational data, we have attempted to capture Mn-oxo species in nanoparticles sample through an in-situ Raman spectroscopy analysis. We were able to observed a broad Raman peak near 744 cm^{-1} when the anodic potential applied to the Ni-Mn_3O_4 sample (Figure 6 and Supplementary Figure 11). The broad Raman peak near 744 cm^{-1} is reversibly affected by the applied potential and is observed only when the potential above the onset for water oxidation, which indicate that the observed peak is from the active intermediate species. The broad Raman peak is red-shifted to 716 cm^{-1} in $H_2{}^{18}O$ electrolyte, while there is no change in the D_2O electrolyte. The observed potential dependency, peak position, and isotope results clearly indicate that the broad Raman peak at 744 cm^{-1} can be assigned to the

stretching vibrational mode of the Mn(IV)-oxo species during the water oxidation reaction. Details are described in the revised manuscript. We also added Figure 6, Supplementary Figure 11, and related discussion as follows:

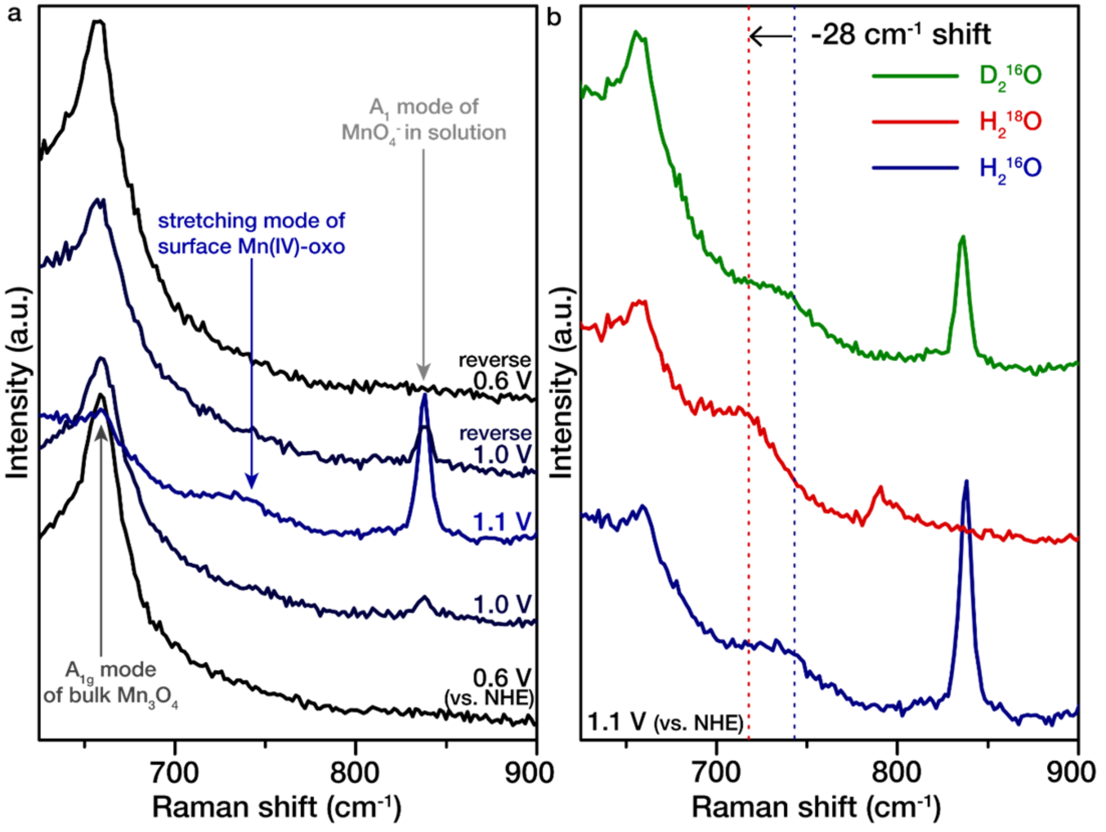

Figure 6 | In-situ Raman analysis. a, Potential dependent in-situ Raman spectra of Ni-Mn_3O_4 NPs/NiO/FTO measured by increasing the applied potential from 0.6 V (vs. NHE) to the above of onset potential (1.1 V vs. NHE) and then decreased to the 0.6 V (vs. NHE). **b**, In-situ Raman spectra during the water oxidation (1.1 V vs. NHE) under various electrolytes. The observed broad Raman peak found in $H_2{}^{16}O$ electrolyte (at 744 cm^{-1}) is red-shifted to 716 cm^{-1} in ^{18}O labeled electrolyte, whereas there is no peak shift in the $D_2{}^{16}O$ electrolyte.

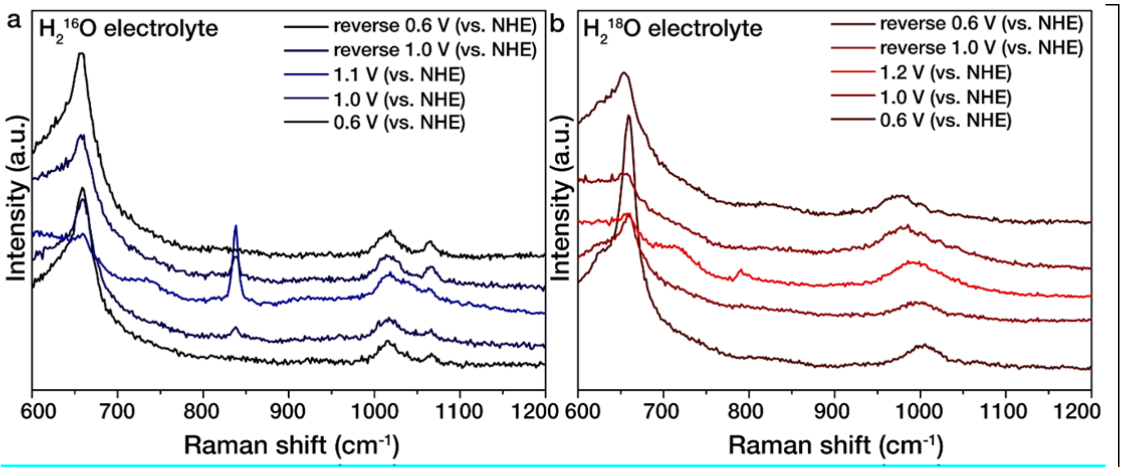

Supplementary Figure 11 | In-situ Raman spectra of Ni-Mn_3O_4 NPs/NiO/FTO. Raman spectra are observed under the 1 M $KHCO_3$ in $H_2{}^{16}O$ (**a**) and 1 M $KHCO_3$ in $H_2{}^{18}O$ (**b**). Applied potential is increased from 0.6 V (vs. NHE) to 1.1 V (vs. NHE) and then reversely decreased to 0.6 V (vs. NHE). Symmetric stretching vibrational mode of C–O from CO_3^{2-} (1066 cm^{-1}) and C–OH from HCO_3^- (1016 cm^{-1}) are clearly observed in the $H_2{}^{16}O$ electrolyte.[R3] In the $H_2{}^{18}O$ electrolyte, only one broad peak from carbonate ion is observed, which is gradually red-shifted by the substitution of ^{18}O occurring as the measurement time passes. Note that, Raman peak at 837 cm^{-1} in $H_2{}^{16}O$ electrolyte can be assigned to soluble MnO_4^-.[R4] The magenta-colored soluble MnO_4^- species are generally observed for manganese-based catalysts when the anodic potential is applied, which could ultimately lead to deactivation of the manganese-based electrodes.[R5]

The description of above figure in manuscript is as below.

Furthermore, potential-dependent in-situ Raman spectroscopy analysis was conducted to get direct evidence for oxo species (Fig. 6 and Supplementary Fig. 11). The sharp and intense Raman peak at 660 cm^{-1}, corresponding to the A_{1g} vibrational mode of spinel crystal structure,[44] is visible at the open circuit potential (Fig. 6a). Note that the Raman peak of soluble MnO_4^- is observed at 837 cm^{-1} before the onset potential (1.0 V vs. NHE).[45] When the anodic potential is above the onset potential for water oxidation reaction (1.1 V vs. NHE), a broad Raman peak near 744 cm^{-1} is developed. This broad Raman peak reversibly disappears when the applied potential is below the onset potential, which indicates that the origin of this Raman peak is from the active intermediate species. From the previously studied molecular complexes, reported Raman peak positions for stretching vibrational mode of Mn(IV)-oxo species are between 707 to 803 cm^{-1},[46-48] while those of Mn(V)-oxo species are between 957 to 997 cm^{-1}.[49-51] Therefore, it is reasonable to conclude that the broad Raman peak is attributed to the stretching vibrational mode of Mn(IV)-oxo species.

To further confirm the nature of the Mn(IV)-oxo species, in-situ Raman spectra are also obtained in the $H_2{}^{18}O$ and $D_2{}^{16}O$ electrolyte (Fig. 6b). The broad Raman peak at 744 cm^{-1} is red-shifted to 716

cm^{-1} in the $H_2{}^{18}O$ electrolyte. The oxygen isotope shift of 28 cm^{-1} with ^{18}O substitution is well-matched with the expected value of 33 cm^{-1} from a diatomic oscillator calculation of Mn(IV)-oxo species. Besides, the positions of Raman peaks remain unchanged in the $D_2{}^{16}O$ electrolyte compared to the spectra obtained in the $H_2{}^{16}O$, which means that the captured intermediate species do not include hydrogen atom. Labeling experimental results clearly prove that the broad Raman band at 744 cm^{-1} can be assigned to the stretching vibrational mode of Mn(IV)-oxo species.

[Reviewer 2-Comment 2] *Second, the catalytic relevance of the low-spin Mn(IV) species is unclear. Is the low-spin Mn(IV) center truly an on-path intermediate or just a species that accumulates at very high potentials? Why should the low-spin Mn(IV) species be better suited for oxygen-evolution than the high-spin analogue?*

[Response]

We appreciate the reviewer for the valuable comments. Considering that the potential-dependent reversible generation/disappearance of EPR and Raman spectroscopic signals, especially for before and after onset potential, captured low-spin Mn(IV)-oxo species would be responsible for the catalytic water oxidation reaction.

We firstly observed the increased current density of Ni-Mn_3O_4 NPs than Mn_3O_4 NPs sample. In addition, the turnover frequency value was derived to compare the intrinsic activity. The number of surface active site was acquired through the electrochemically active surface area (ECSA) measurement. Ni-Mn_3O_4 NPs sample (0.0325 s^{-1} at 1.3 V) exhibited slightly increased turnover frequency value compared to Mn_3O_4 NPs sample (0.0279 s^{-1} at 1.3 V), which indicates favorable participation of low-spin Mn(IV)-oxo species for catalytic turnover. We could find the calculation result that the low-spin Mn(IV)-oxo (S=1/2) has lower energy barriers for the hydrogen atom transfer (HAT) reaction than high-spin Mn(IV)-oxo [*Chem. Commun.* 51, 13094 (2015)]. However, at the current stage, there is no theoretical results about the reactivity of low-spin Mn(IV)-oxo in water oxidation reaction. Further study is needed to point out the exact favorable role of low-spin Mn(IV)-oxo species during the complex water oxidation pathway in heterogeneous catalysts.

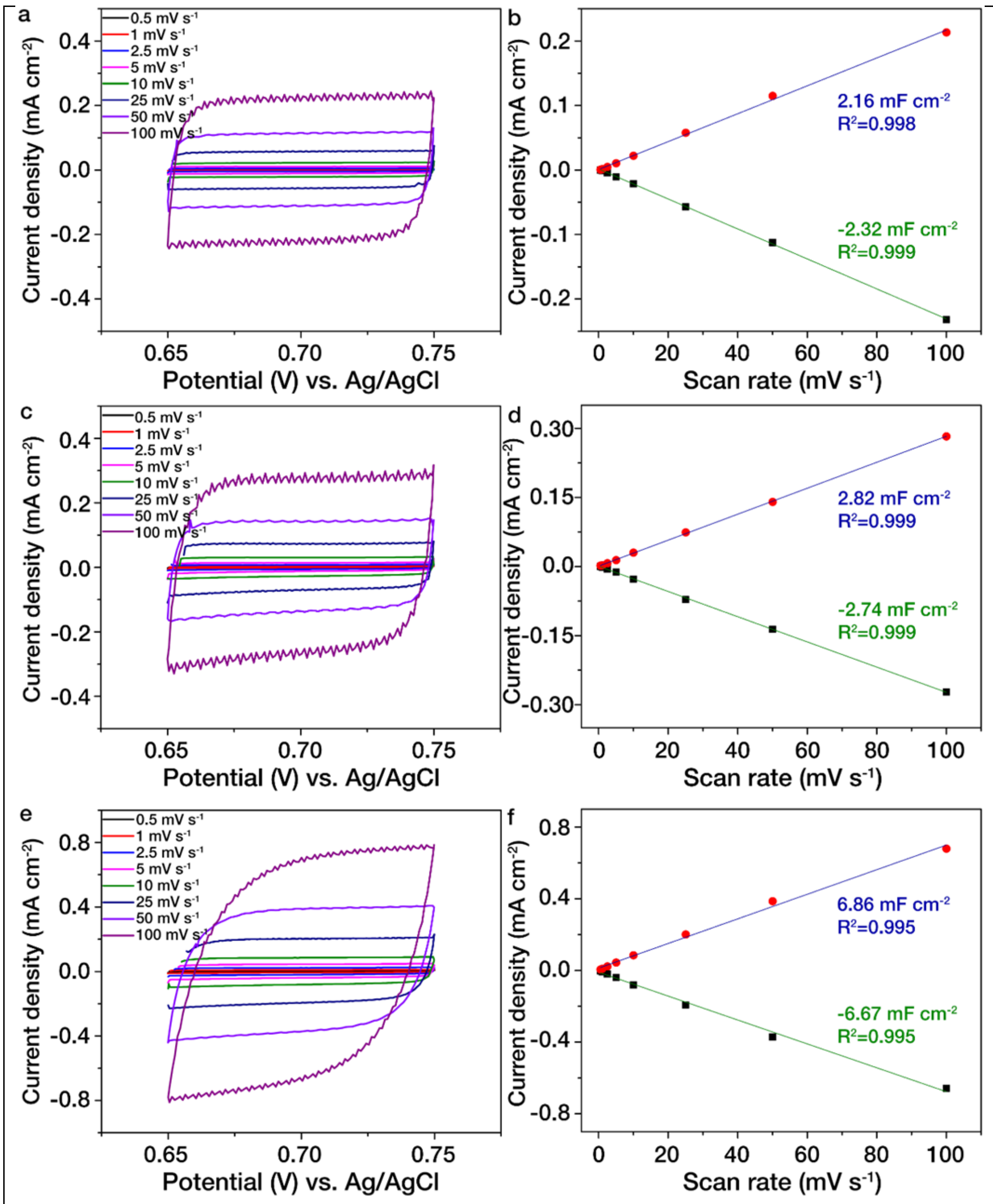

Supplementary Figure 1 | The electrochemically active surface area of prepared electrodes. Cyclic voltammograms of NiO/FTO (**a**), Mn_3O_4 NPs/FTO (**c**), Ni-Mn_3O_4 NPs/NiO/FTO (**e**) in 0.5 M phosphate buffer (pH 7). Plots of the anodic and cathodic current densities versus scan rates (**b**: NiO/FTO, **d**: Mn_3O_4 NPs/FTO, and **f**: Ni-Mn_3O_4 NPs/NiO/FTO). The double-layer capacitance is derived from the slops of linear fitting results.

	C_{dl} (mF)	ECSA (cm^2)	i at 1.3 V (mA)	number of active sites (Mn site cm^{-2})	Turnover frequency at 1.3 V (s^{-1})
Ni-Mn_3O_4 NPs/NiO/FTO	3.38	84.6	1.995	1.134E15	0.0325
Mn_3O_4 NPs/FTO	1.39	34.8	0.705	1.134E15	0.0279
NiO/FTO	1.12	28.0	0.105	1.444E15	0.00405

Supplementary Table 1 | Calculated turnover frequency for prepared electrodes. The electrochemically active surface area is calculated from measured double-layer capacitance. The number of active sites is calculated using the crystallographic data of Mn_3O_4 for Ni-Mn_3O_4 NPs/NiO/FTO and Mn_3O_4 NPs/FTO samples, and NiO for NiO/FTO sample. The unit cell of Mn_3O_4 contains 12 Mn atoms and the volume 314.3 Å^3. The calculated number of surface Mn atoms is 1.134×10^{15} Mn atoms cm^{-2}.[R6] The unit cell of NiO contains 4 Ni atoms and the volume 72.87 Å^3. The calculated number of surface Ni atoms is 1.444×10^{15} Ni atoms cm^{-2}.[R7] Turnover frequencies at 1.3 V (vs. NHE) are calculated by the following equation with the assumption that all metal atoms on the surface serve as the active sites.

$$\text{turnover frequency} = \frac{(current\ density\ at\ 1.3\ V\ vs.NHE)}{4 \times F \times (number\ of\ active\ sites)}$$

[Reviewer 2-Comment 3] *In the abstract it is stated that the low-spin Mn(IV) signal evolves during the "stepwise transition from the Jahn-Teller-distorted Mn(III)." In addition, on line 163, the authors state that the Mn(III) signal slightly decreases as the anodic potential increased. While the low-spin Mn(IV) signal clearly increases in intensity as the potential is increased, there does not appear to be a concomitant decrease in the Mn(III) EPR signal. To demonstrate that one species converts to the other, as stated in the abstract, the Mn(IV) and Mn(III) signals should be quantified (by integration) and this quantity should be plot versus the applied potential. This plot could provide evidence if the Mn(III) is being consumed as the low-spin Mn(IV) is being formed.*

[Response]

We thank the reviewer for the valuable comments. In fact, we made a mistake in plotting the EPR data in the main figure; the intensities of each spectrum were modified for visibility. Now in the revised manuscript, we plotted the data without the intensity modification (Figure 3). Besides, as the reviewer suggested, we added the plot of intensity changes of the Mn EPR signal for each oxidation state depending on the applied potentials in Supplementary Figure 5 to clearly demonstrate the one species converts to the other as the applied potential increases. We thought that revised Figures provides evidence that the Mn(III) is being consumed as the low-spin Mn(IV) is being formed.

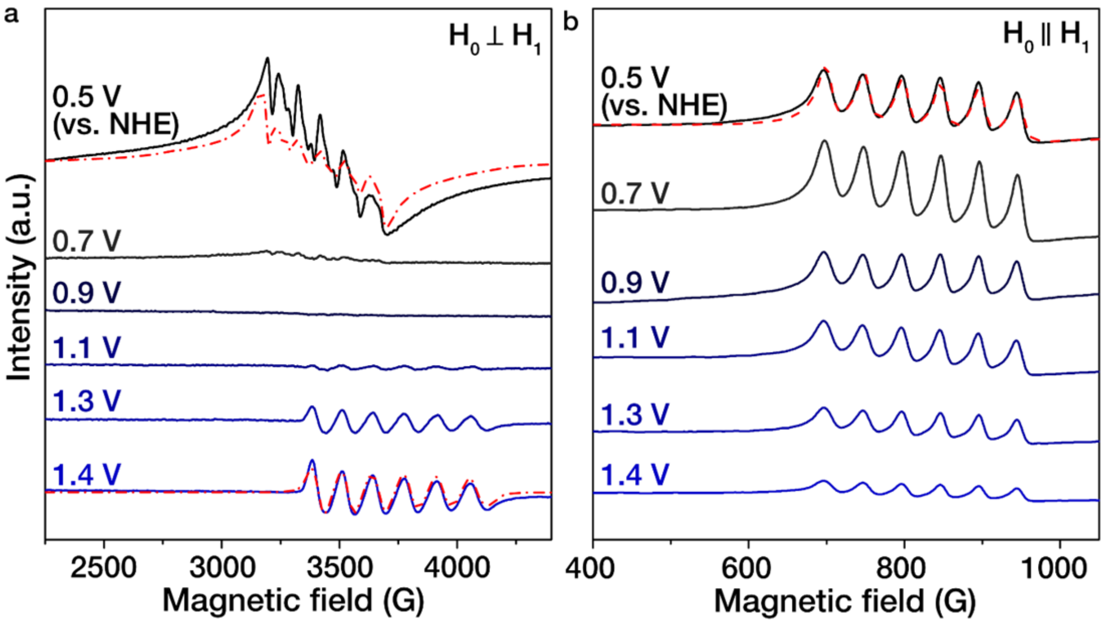

Figure 3 | Measured and simulated potential dependent EPR spectra. **a** and **b**, Potential-dependent X-band CW-EPR spectra of Ni-Mn_3O_4 NPs/NiO in perpendicular (**a**) and parallel (**b**) modes. The dashed red lines are spectral simulations that are well matched with the experimental curves.

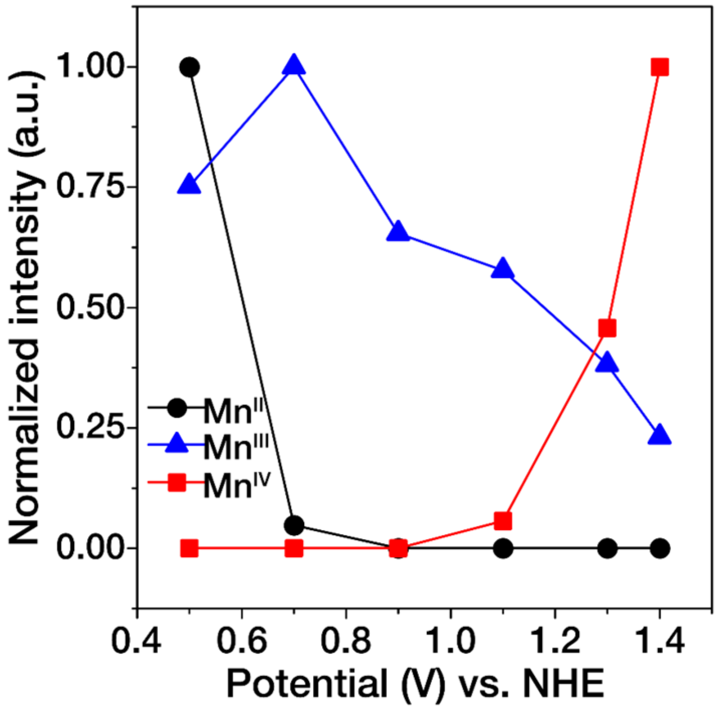

Supplementary Figure 5 | Normalized intensities of each Mn species depending on the applied potential.

[Reviewer 2-Comment 4] *The computational work is not completely described. First, the authors have used computations to model the Mn(IV)-oxo species as an octahedral complex, but the most relevant point of comparison (the low-spin Mn(IV)-nitride) is a tetrahedral complex. Why was a tetrahedral geometry not considered?*

[Response]

We thank the reviewer for the valuable comments. According to the reported low-spin Mn(IV) in tetrahedral geometry (*g* = [2.35, 1.973, 1.965], |A| = [300, 74, 202] MHz), EPR signal is largely different from the EPR signal that we experimentally observed. Additionally, it was reported that the Mn(III) ions located in a tetrahedral environment of Mn_3O_4 are kinetically stable without further oxidation to Mn(IV) under the electrochemical oxidation potential [*Proc. Natl. Acad. Sci. U.S.A.* 115 (23), E5261 (2018)]. Consequently, we considered only the octahedral geometry for Mn(IV) species between two possible octahedral and tetrahedral geometries existing in the Mn_3O_4 crystal structure.

[Reviewer 2-Comment 5] *Second, while A values were calculated, g values were not. Given the highly unusual g values observed experimentally, it is odd that the g values were not calculated and compared with experiment.*

[Response]

By following the reviewer's comment, we included g values calculated using DFT. We obtained DFT-calculated *g*-values of ***g*** = [1.993, 1.998, 2.000] for high-spin Mn(IV), and ***g*** = [1.967, 2.006, 2.007] for low-spin Mn(IV). Although the DFT-calculated g_{xx} component exhibits a smaller value than 2, the calculated *g*-values does not fully explain the experimentally observed *g*-values. DFT-calculated *g*-values are known to be quantitatively erroneous for near degenerate systems, mostly due to the limitation of single-reference calculations, which can be improved by employing computationally demanding but more accurate multi-reference calculation methods such as CASSCF/NEVPT2 [*J. Am. Chem. Soc.* 141, 2421 (2019)]. We would like to point out that quantitatively reliable modelling of ligand field strength is extremely difficult for the metal atom embedded in the inorganic nanoparticle lattice, unlikely to the organometallic system. This renders a quantitative comparison between experimental and computational results. In the current study, we showed that DFT calculation provides the origin of distortion, although there is some limitation in

exact g value. Based on this understanding, we will develop the theoretical model to quantify *g*-value in inorganic bulk crystals. That will be an important research direction in future research.

[Reviewer 2-Comment 6] *Third, the metric parameters for the low-spin Mn(IV) complex are of interest. The authors should discuss bond lengths, angles, etc., especially in relation to the high-spin counterpart. Do the computations offer insight into the structural basis for the stabilization of the low-spin state?*

[Response]

By following the reviewer's comment, we summarized important metrics about the local structure near Mn center by comparing the DFT-optimized structure of bare Mn_3O_4 and that of Ni-Mn_3O_4 in Supplementary Figure 13. Despite of some quantitative differences, Ni substitution decreases the axial Mn–O distance and increase the rhombicity, both of which can stabilize the low-spin state.

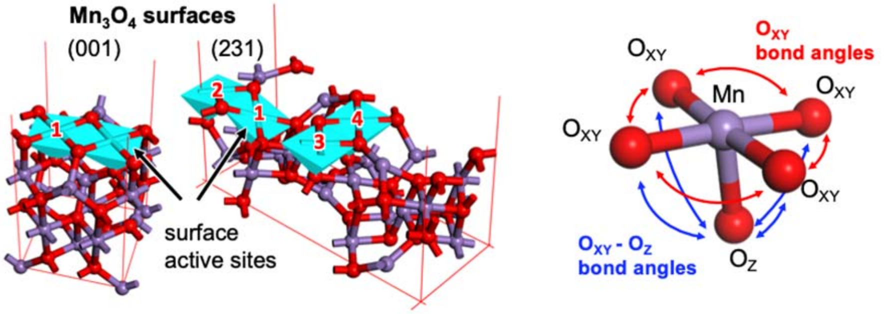

	Mn-O_{XY} distances (Å)				Mn-O_Z distance (Å)	O_{XY}-Mn-O_{XY} angles (°)				O_{XY}-Mn-O_Z angles (°)			
(001)	1.957	1.957	1.924	1.924	2.301	98.67	98.67	81.38	81.28	97.35	97.35	82.35	82.35
(001)-Ni	1.996	1.924	1.924	1.918	2.170	98.30	96.27	82.89	82.68	97.65	96.07	86.70	79.56
$(231)_1$	1.973	1.955	1.954	1.910	2.316	97.73	95.67	84.85	81.89	105.67	103.97	77.02	75.84
$(231)_1$-Ni	2.000	1.978	1.930	1.926	2.215	103.76	87.40	84.79	83.96	105.10	103.72	80.59	79.54
$(231)_2$	2.135	1.986	1.912	1.892	2.127	97.19	93.93	85.13	83.59	100.88	98.85	85.82	82.50
$(231)_2$-Ni	2.199	1.979	1.961	1.897	2.076	95.91	95.48	86.37	82.16	98.40	96.69	84.81	84.38
$(231)_3$	2.068	2.019	2.009	1.977	2.175	103.74	97.95	79.50	78.88	98.94	97.68	88.86	80.35
$(231)_3$-Ni	2.080	2.029	1.989	1.974	2.141	104.67	97.77	79.22	78.09	97.42	96.87	89.16	85.18
$(231)_4$	2.077	2.068	2.013	1.971	2.138	102.97	100.00	79.80	77.37	96.74	93.89	91.27	81.86
$(231)_4$-Ni	2.071	2.053	2.015	1.966	2.084	103.74	100.18	79.52	76.45	98.83	93.74	93.73	81.93

Supplementary Figure 13 | Calculated bond distances and angles of truncated surface Mn active sites before and after the Ni substitution. Ni substituted structures were chosen from the

maximally compressed axial Mn–O bond cases in Figure 6c.

[Reviewer 2-Minor comment 1] *The oxygen-evolving complex is more commonly referred to as the Mn4Ca cluster (as is done in the abstract), rather than the MnCa cluster, to denote the Mn nuclearity.*

[Response]

Thank you for your helpful comments. We agree that the suggested expression would be more appropriate. By following the reviewer's suggestion, we changed the expression to Mn_4Ca cluster in the revised manuscript.

[Reviewer 2-Minor comment 2] *The assignment of the 400 nm and 650 nm electronic absorption features for the low-spin Mn(IV) species to LMCT and d-d transitions, respectively, seems at odds with the relative intensities of these features. In fact, the intensities are inverted compared to that expected and compared to that observed for the intensities of the LMCT and d-d transitions for the putative high-spin Mn(IV)-oxo species shown in Figure 2e. What explanation is there for the big change in the relative intensities of these features?*

[Response]

Thank you for pointing out an important part that was lacking in explanation. We agree that the previous description was not enough to describe the change of our electrode. The broad peak between 550 and 800 nm is due to the overlapping between the low-spin Mn(IV)-oxo's another LMCT band and the absorption of nickel oxyhydroxide formed by the oxidation of underlying NiO layer. From the calculation result, which will be discussed in response for minor comment 4, low-spin Mn(IV)-oxo exhibits another intense LMCT band at 554.1 nm. Additionally, bottom NiO layer is oxidized to nickeloxyhydroxide under the anodic potential applied condition, which contributes broad absorption from 500 to 750 nm (Figure R1).

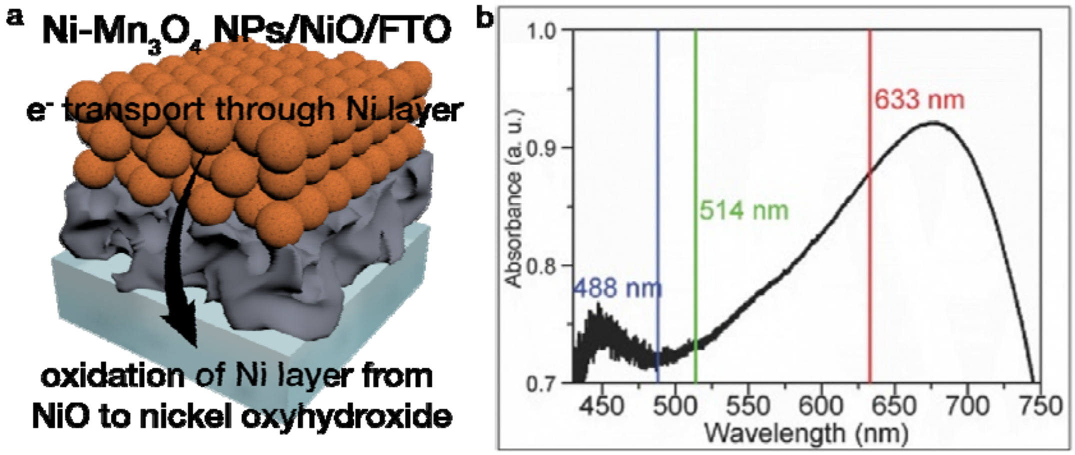

Figure R1 | a, Schematic illustration of electron transport under the anodic potential applied condition. **b**, UV-Vis spectrum of nickel oxyhydroxide (adapted from reference [*Adv. Sci.*, 3, 1500433 (2016)]).

Clearly, Ni-Mn_3O_4 NPs/NiO catalyst exhibits a shifted peak at 420 nm (denoted as # in Fig. 2f) and an intense absorption change between 550 to 800 nm region (denoted as an arrow in Fig. 2f). The shifted peak at 420 nm is originated from new intermediate species from Ni-Mn_3O_4 NPs layer. The calculation results, which will be discussed later, support that the origin of the red-shifted peak at 420 nm is a π-type LMCT band of the new intermediate species. It is turned out that the broad peak between 550 and 800 nm is due to the overlapping between the new intermediate's another LMCT band and the absorption of nickel oxyhydroxide formed by the oxidation of underlying NiO layer[32,33].

[Reviewer 2-Minor comment 3] *For the more general audience of Nature Communications, the Vvib and VL terms should be more completely described. In addition, the EJPT term (line 224) should be defined more explicitly. I presume this is the splitting between the xz and yz orbitals, but this is not stated.*

[Response]

We appreciate your kind suggestions. We added more description for each terms. Shown below is the revised discussion that has been added to the manuscript.

The *g*-values can be described in terms of the ratio of the sum of the vibronic coupling energy at the equilibrium distortion (V_{vib}) and the solvent-dependent environmental energies affecting the orbital splitting (V_L) to the spin-orbit coupling constant (λ),[39] through the fictitious angle 2θ:

In addition, the PJT splitting energy (E_{PJT}), which indicates the stabilization resulted from the breaking of the orbital degeneracy, can be estimated from the *r* value and *g*-values as $E_{PJT} = \lambda[1 + \tan^2 2\theta]^{1/2} = 1000\ cm^{-1}$ by using equations (1) to (3).

[Reviewer 2-Minor comment 4] *The spectral assignments from the TD-DFT computations, as LMCT or d-d bands, are incomplete. The computations provide precise information on the orbitals involved in these excitations, and this information should be provided. For example, the S = ½ Mn(IV) ion will have many d-d transitions. Which are observed in the visible region for the TD-DFT computations? Are the LMCT transitions of pi- or sigma-type?*

[Response]

We appreciate for the reviewer's careful comment. By following the reviewer's comment, we analysed the electron density difference map for the transitions with the large oscillatory strength near 400 nm and 600 nm.

For the transitions near 400 nm, both high-spin and low-spin cases show the LMCT character of π-type. For the transitions near 600 nm, the high-spin Mn(IV)-oxo shows the *d*-*d* transition at 534.5 nm in consistent with the previous report [*J. Am. Chem. Soc.* 139, 2277 (2017)]. Interestingly, however, the low-spin Mn(IV)-oxo shows the LMCT character of π-type at 554.1 nm. Due to the broken degeneracy of d_{xz} and d_{yz} orbital, which form π and π* orbitals by hybridizing with p_x and p_y orbitals, low-spin Mn(IV)-oxo can have different energies for the π-type LMCT. Therefore, low-spin Mn(IV)-oxo shows a relatively intense peak near 600 nm compared to the *d*-*d* transition peak of high-spin Mn(IV)-oxo.

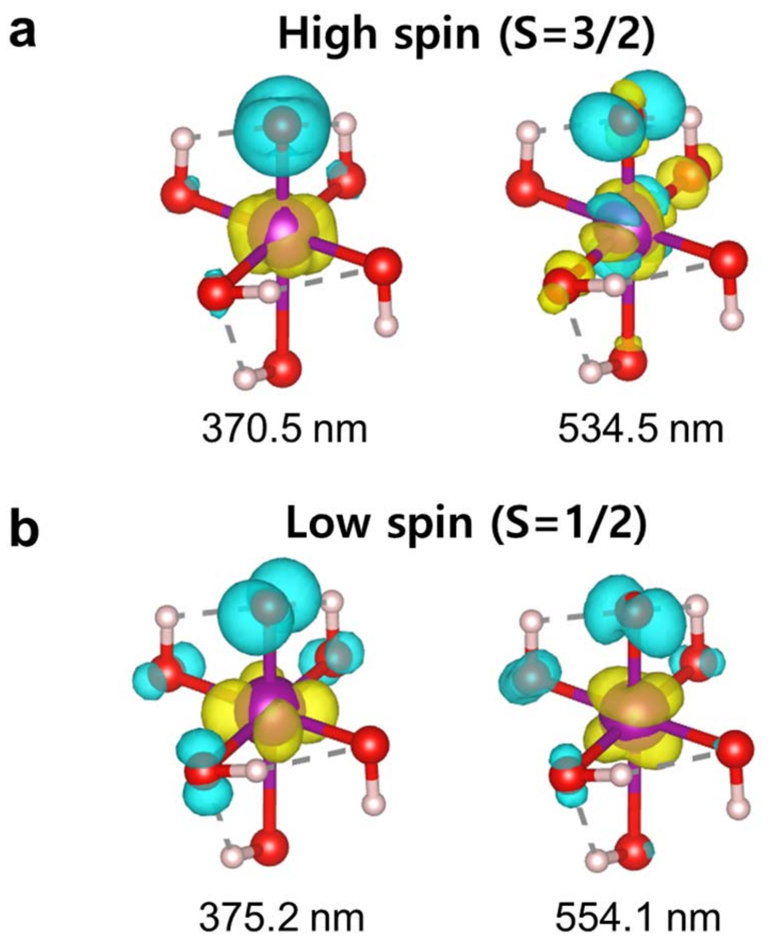

Supplementary Figure 17 | Electron density difference map for the transitions with the large oscillatory strength near 400 nm and 600 nm. a, High-spin Mn(IV)-oxo shows π-type ligand-to-metal charge transfer (LMCT) band at 370.5 nm and *d*-*d* transition band at 534.5 nm. **b,** Low-spin Mn(IV)-oxo shows π-type LMCT bands at 375.2 nm and 554.1 nm. Due to the broken degeneracy of d_{xz} and d_{yz} orbital, which form π and π* orbitals by hybridizing with p_x and p_y orbitals, low-spin Mn(IV)-oxo can have different energies for the π-type LMCT.

The description of above figure in manuscript is as below.

Using the same cluster models, we further calculated the UV-Vis spectrum of Mn(IV)-oxo species using time-dependent DFT (Fig. 7d and Supplementary Fig. 17). As shown in Fig. 7d, the π-type LMCT band for the high-spin Mn(IV)-oxo is found at 370.5 nm, while that for the low-spin Mn(IV)-oxo is found to be redshifted to 375.2 nm, which agrees with our experimentally measured UV-Vis spectrum. In addition, the high-spin Mn(IV)-oxo exhibits a *d*-*d* transition band at 534.5 nm, while the low-spin Mn(IV)-oxo exhibits another LMCT band at 554.1 nm due to the broken degeneracy of d_{xz} and d_{yz} orbital. Therefore, low-spin Mn(IV)-oxo shows a relatively intense

absorption peak at 554.1 nm compared to high-spin Mn(IV)-oxo.

[Reviewer 2-Minor comment 5] *The [e3] and [b2e1] nomenclature (using subscripts) is used throughout the paper to represent electron configurations. However, the standard nomenclature is to use superscripts to note orbital occupancy; i.e., (e)3 and (b)2(e)1 (using superscripts). This approach has the advantage that a (b)2 configuration can be distinguished from a b2 Mulliken symbol.*

[Response]

We appreciate your kind suggestions. By following the reviewer's suggestion, we changed the expression in the revised manuscript.

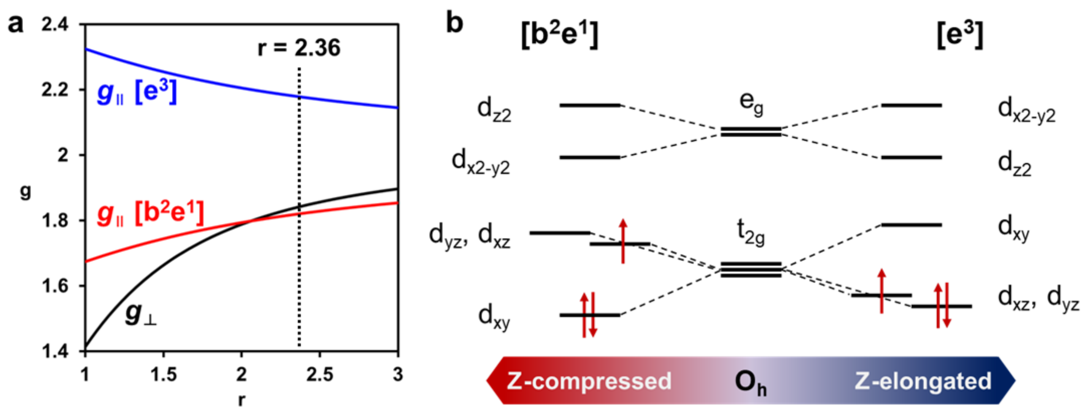

Figure 5 | Evaluation of *g*-values and corresponding *d*-orbital configurations of the Mn(IV) species. a, Plot of *g*-values for the [b^2e^1] and [e^3] configurations as a function of ***r*****. b**, Qualitative *d*-orbital splitting pattern for tetragonally compressed ([b^2e^1] configuration) and elongated ([e^3] configuration) low-spin octahedral Mn(IV) centers.

Reviewer 3

In this very interesting manuscript, the authors report for the first time on new intermediates during the water oxidation reaction of manganese-oxide based electrocatalysts, assigned as alow-spin Mn(IV)-oxo species. To capture the reactive intermediate, they have used several characterization techniques, such as: in-situ potential dependent UV-Vis spectroscopy, ex-situ EPR under electrocatalytic operation conditions, and DFT calculations. These important findings should pave the way to widen our understanding regarding the mechanisms of electrochemical water oxidation. In addition, the gained insights will aid in the future design of highly efficient catalysts for a wide range of applications. As such, I recommend publication in Nature Communications, subject to addressing several points.

[Response]

We really appreciate your high valuation and positive comments on our manuscript. According to your suggestion, the logic of the introduction and the electrochemical activity comparison part, in particular, were revised more clearly. Additionally, in-situ Raman results were added during the revision to provide direct evidence for the generation of terminal Mn(IV)-oxo species. The detailed point-by-point responses are below.

[Reviewer 3-Comment 1] *The authors should discuss the reasons behind the motivation to run water oxidation reaction at neutral pH, rather than in alkaline solutions where known catalysts exhibit much higher catalytic activity*

[Response]

We appreciate the insightful comments from the reviewer. As the reviewer points out, most man-made electrocatalysts exhibit higher catalytic activity for water oxidation reaction under the alkaline pH than neutral pH conditions. However, the biological system (Mn_4Ca cluster) efficiently catalyzes water oxidation reaction under mild condition (pH of 6.5) using only Mn and Ca metal atoms. The unique capabilities of the biological system have inspired researchers to develop efficient water oxidizing catalysts under the neutral condition. Recently, much interest in water oxidation under the neutral pH condition has been increased based on several advantages as well as scientific interests. Firstly, a neutral pH condition is more environmentally friendly with fewer corrosion issues, reducing the cost of electrolyzer cells. Additionally, efficient water oxidizing catalysts under neutral pH would

facilitate the combination with CO_2 reduction or microbial electrolysis cell. Following the reviewer's suggestion, we briefly discuss the motivation for neutral performing water oxidation catalysts. Shown below is the revised discussion that has been added to the manuscript.

The proposed mechanistic cycle of water oxidation reaction starts with the adsorption of a water molecule on the surface of transition metal oxide catalysts. Then, the adsorbed water molecule undergoes multiple protons and electrons transfer with a redox state change of the transition metal site to generate high-valent transition metal-oxo species involved in the O–O bond formation step. Understanding each elementary step and developing catalysts under neutral pH conditions have been recently getting much interest due to the advantage of environmentally friendly, less corrosive electrolyte and the possibility of combining with CO_2 reduction or microbial hybrid electrolysis cell.[5-7]

[Reviewer 3-Comment 2] *Is there any other possible characterization tools that could be utilized in the future for catalytic intermediate capture?*

[Response]

In this manuscript, we performed X-ray absorption near edge structure (XANES), UV-Vis, and EPR spectroscopy analysis during the water oxidation reaction to characterize intermediate species. We also added in-situ Raman analysis results that contain the vibrational information of intermediate species. In-situ infrared spectroscopy is another versatile technique to get vibrational data. Luminescence and Mössbauer spectroscopy could also be utilized in the characterization of intermediate species. X-ray techniques, such as X-ray diffraction and extended X-ray absorption fine structure, are useful to get the structural information of electrocatalysts during the reaction. In addition to capture the signal of intermediate species at the steady state condition, further time-resolved spectroscopic analysis could provide kinetic information of detected intermediate species. Besides, surface interrogation scanning electrochemical microscopy technique directly provides the electrochemically active surface-site density that is necessary to calculate turnover frequency of heterogeneous electrocatalysts.

[Reviewer 3-Comment 3] *Can the authors compare the water oxidation intrinsic activity (TOF) for their characterized samples (Mn3O4, Ni-Mn3O4, and NiO)?*

[Response]

We thank the reviewer for the valuable comments. As the reviewer suggested, turnover frequency (TOF) values are obtained by dividing the measured current by the number of active sites. The number of active sites is determined by measuring the electrochemically active surface area with the assumption that all metal sites on the surface participate in the reaction. TOF value of only NiO sample shows a much lower value than manganese samples, indicating superior electrocatalytic activity of Mn_3O_4 NPs and Ni-Mn_3O_4 NPs under neutral pH conditions. Additionally, Ni-Mn_3O_4 NPs/NiO sample exhibits a larger TOF value than Mn_3O_4 NPs. We added this data in the supplementary information.

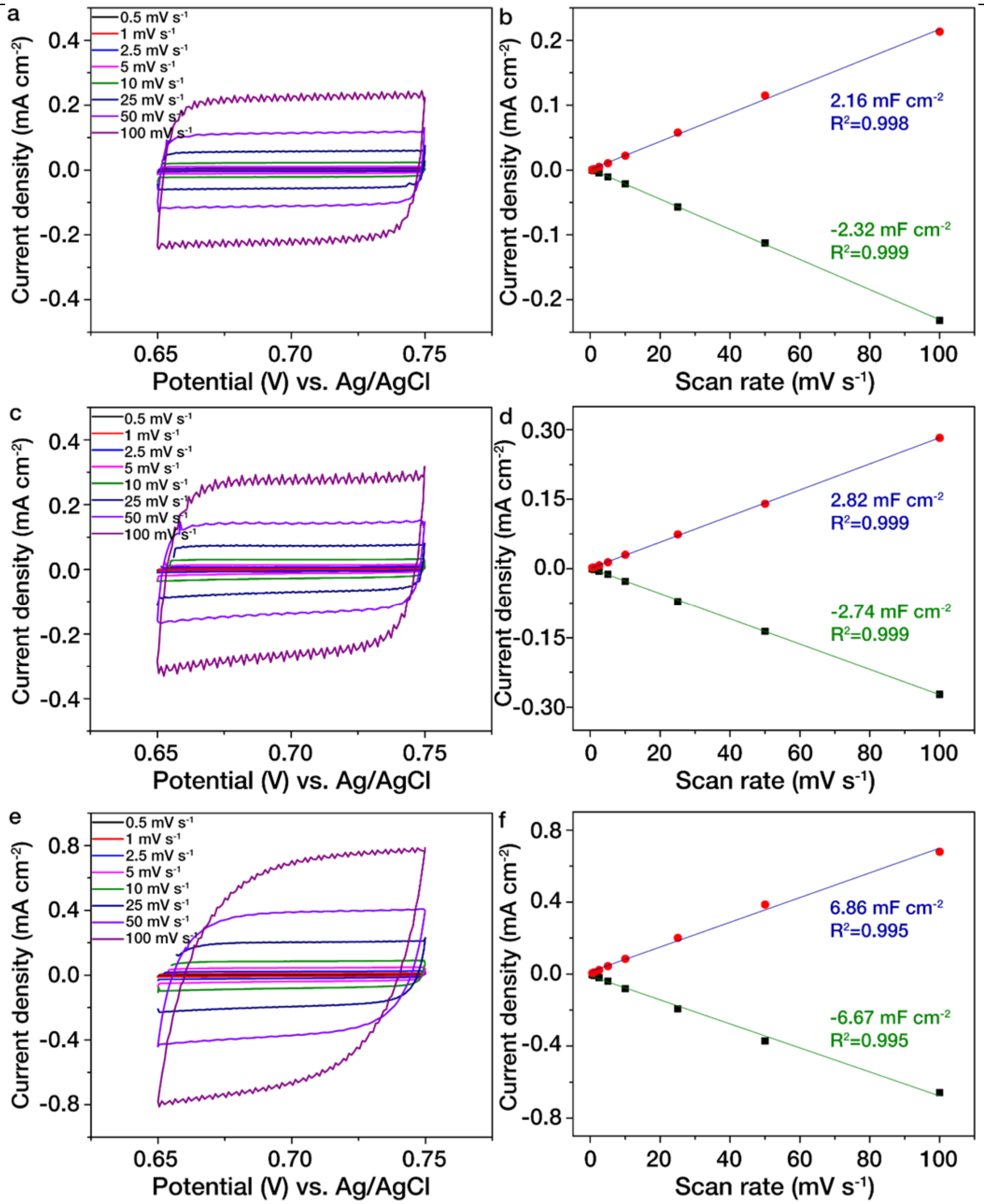

Supplementary Figure 1 | The electrochemically active surface area of prepared electrodes. Cyclic voltammograms of NiO/FTO (**a**), Mn_3O_4 NPs/FTO (**c**), Ni-Mn_3O_4 NPs/NiO/FTO (**e**) in 0.5 M phosphate buffer (pH 7). Plots of the anodic and cathodic current densities versus scan rates (**b**: NiO/FTO, **d**: Mn_3O_4 NPs/FTO, and **f**: Ni-Mn_3O_4 NPs/NiO/FTO). The double-layer capacitance is derived from the slops of linear fitting results.

	C_{dl} (mF)	ECSA (cm^2)	i at 1.3 V (mA)	Number of active sites (Mn sites cm^{-2})	Turnover frequency at 1.3 V (s^{-1})
Ni-Mn_3O_4 NPs/NiO/FTO	3.38	84.6	1.995	1.134E15	0.0325
Mn_3O_4 NPs/FTO	1.39	34.8	0.705	1.134E15	0.0279
NiO/FTO	1.12	28.0	0.105	1.444E15	0.00405

Supplementary Table 1 | Calculated turnover frequency for prepared electrodes. The electrochemically active surface area is calculated from measured double-layer capacitance. The number of active sites is calculated using the crystallographic data of Mn_3O_4 for Ni-Mn_3O_4 NPs/NiO/FTO and Mn_3O_4 NPs/FTO samples, and NiO for NiO/FTO sample. The unit cell of Mn_3O_4 contains 12 Mn atoms and the volume 314.3 Å^3. The calculated number of surface Mn atoms is 1.134×10^{15} Mn atoms cm^{-2}.[R6] The unit cell of NiO contains 4 Ni atoms and the volume 72.87 Å^3. The calculated number of surface Ni atoms is 1.444×10^{15} Ni atoms cm^{-2}.[R7] Turnover frequencies at 1.3 V (vs. NHE) are calculated by the following equation with the assumption that all metal atoms on the surface serve as the active sites.

$$\text{turnover frequency} = \frac{(current\ density\ at\ 1.3\ V\ vs.NHE)}{4 \times Faraday\ constant \times (number\ of\ active\ sites)}$$

The description of above figure and table in manuscript is as below.

Ni-Mn_3O_4 NPs/NiO sample exhibits a higher turnover frequency compared to Mn_3O_4 NPs sample, which is calculated by using the measured electrochemically active surface area (Supplementary Fig. 1 and Supplementary Table 1).

Reviewer #2 (Remarks to the Author):

The authors have done an exceptional job of revising the manuscript. All of my comments were responded to in a thoughtful and comprehensive manner. My main concern regarding the initial submission was the lack of vibrational data to justify the Mn(IV)-oxo assignment. The in-situ Raman data in the revised version has set these concerns to rest. I support publication of this revised manuscript.

Timothy A. Jackson

Reviewer #3 (Remarks to the Author):

The Authors have now fully and carefully addressed all questions/comments raised by myself and the other reviewers.
Specifically, the modified introduction section, addition of TOF values to compare the intrinsic activity of all samples, and the addition of in-situ Raman data, served to further improve the manuscript's quality.
Hence, I now recommend publication in Nature Communications without further revision.

Response to reviewers

Reviewer 2

The authors have done an exceptional job of revising the manuscript. All of my comments were responded to in a thoughtful and comprehensive manner. My main concern regarding the initial submission was the lack of vibrational data to justify the Mn(IV)-oxo assignment. The in-situ Raman data in the revised version has set these concerns to rest. I support publication of this revised manuscript.

[Response]

We appreciate the reviewer's valuation, and support on publication of the revised manuscript.

Reviewer 3

The Authors have now fully and carefully addressed all questions/comments raised by myself and the other reviewers. Specifically, the modified introduction section, addition of TOF values to compare the intrinsic activity of all samples, and the addition of in-situ Raman data, served to further improve the manuscript's quality. Hence, I now recommend publication in Nature Communications without further revision.

[Response]

We really appreciate your high valuation, and recommendation of publication without further revision.
